# The Contributions of Interlocking Loops and Extensive Nonlinearity to the Properties of Circadian Clock Models

**DOI:** 10.1371/journal.pone.0013867

**Published:** 2010-11-30

**Authors:** Treenut Saithong, Kevin J. Painter, Andrew J. Millar

**Affiliations:** 1 Department of Biological Sciences, Institute of Molecular Plant Sciences, University of Edinburgh, Edinburgh, United Kingdom; 2 Department of Mathematics and Maxwell Institute for Mathematical Sciences, School of Mathematical and Computer Sciences, Heriot-Watt University, Edinburgh, United Kingdom; 3 Centre for Systems Biology at Edinburgh, Edinburgh, United Kingdom; University of Nottingham, United Kingdom

## Abstract

**Background:**

Sensitivity and robustness are essential properties of circadian clock systems, enabling them to respond to the environment but resist noisy variations. These properties should be recapitulated in computational models of the circadian clock. Highly nonlinear kinetics and multiple loops are often incorporated into models to match experimental time-series data, but these also impact on model properties for clock models.

**Methodology/Principal Findings:**

Here, we study the consequences of complicated structure and nonlinearity using simple Goodwin-type oscillators and the complex Arabidopsis circadian clock models. Sensitivity analysis of the simple oscillators implies that an interlocked multi-loop structure reinforces sensitivity/robustness properties, enhancing the response to external and internal variations. Furthermore, we found that reducing the degree of nonlinearity could sometimes enhance the robustness of models, implying that *ad hoc* incorporation of nonlinearity could be detrimental to a model's perceived credibility.

**Conclusion:**

The correct multi-loop structure and degree of nonlinearity are therefore critical in contributing to the desired properties of a model as well as its capacity to match experimental data.

## Introduction

Circadian clocks are the endogenous 24h timing system of living organisms and are believed to be formed from a group of genes and their proteins connected in negative feedback loops [1]. The circadian clock has been studied in a range of organisms across the taxonomic classes [1], [2] from *Synechococcus elongatus* (unicellular cyanobacterium) [3], [4], *Neurospora crassa* (fungus) [5]–[7], *Drosophila melanogaster* (insect) [8]–[11], *Arabidopsis thaliana* (plant) [12]–[15] to mammals [16]–[18]. Despite the apparently independent evolution of circadian clocks within diverse organisms, certain characteristics are shared across all circadian clocks, including the ability to: (1) generate a circa 24h rhythm that is robust to the external/internal variations, and (2) be entrained by rhythmic environmental signals (light-dark cycle or temperature cycle) [1], [2]. The availability of time-series data in mutant organisms, combined with varying input signals, has led to a series of detailed circadian clock models for these organisms (*e.g.* [9], [19]), including Arabidopsis.

The first Arabidopsis clock model, denoted ‘one-loop’ (Figure S1a), was constructed according to the results and hypothesis of Alabadi *et al* (2001) [12], . It is a single negative feedback loop model consisting of two redundant genes encoding MYB transcription factors, *LATE ELONGATED HYPOCOTYL* (*LHY*) and *CIRCADIAN CLOCK ASSOCIATED 1* (*CCA1*), and a gene encoding a pseudo-response regulator protein, *TIMING OF CAB EXPRESSION 1* (*TOC1*). A second ‘two-loop’ model (Figure S1b) was developed to explain the results from mutant plants, especially the *lhy;cca1* double mutant [20], [21]. A hypothetical component ‘*Y*’ forms an additional loop that interlocks with the original one-loop model. Several studies into the functions of *PRR7* (*PSEUDO-RESPONSE REGULATOR 7*) and *PRR9* (*PSEUDO-RESPONSE REGULATOR 9*) in the circadian clock [22]–[24] led to two extended models, a ‘three-loop’ (Figure S1c) [25] and a ‘four-loop’ [26] model. The dissimilar manner in which new components are added to the models differentiate their features, including complexity, robustness and the adaptability to match plant behaviours [25], [26].

The identification of the ‘best’ model is generally evaluated only from its capacity to generate simulated behaviour that fits the experimental data. However, this basic criterion may not sufficiently discriminate the most plausible model. Matching the properties of the model to the nature of the real system could be a second rational criterion [27]. The *model behaviours* correspond to observable rhythms produced by model simulation, such as mRNA expression profiles. *Model properties* are intrinsic characteristics of the model, such as robustness or sensitivity [28].

Complicated models are often built to recapitulate complex dynamic data. It may also be desirable to include many of the observed biochemical processes [9], [19], [25]. The main complexities are intricate circuit structures, nonlinear kinetics, the number of model components and the redundancy of component linkage [28], [29]. However, the complexity affects not only the model behaviours but also a variety of model properties, for instance the adaptability (the ability to replicate the observed behaviours in diverse conditions) and the sensitivity of the model [30], [31]. Increasing complexity may improve the adaptability of the model to fit more of the existing data [30], but simultaneously boost the sensitivity of the model [32].

Robustness is a remarkably important model property indicating the capability to maintain a model behaviour (such as an equilibrium state) in varied conditions [33]–[35], while sensitivity can be defined as the inverse of robustness and is required to sense and respond to perturbations. Both are required for circadian clocks [36]–. Since a trade-off between sensitivity and robustness is a key feature of homeostasis and may be vital for the survival of organisms [33], [34], these properties have been used for validating models of biological processes [27]. The more plausible model was defined as having greater robustness to variations [33], [38]–[40]. Robustness (or sensitivity) is commonly evaluated as the change in model behaviour under a range of parameter changes, though changes in circuit topology or mutational effects may also be tested. Mathematical measures of parameter sensitivity vary considerably, depending on the model behaviour and on the selection of parameter sets. Local analysis may simply test a particular fold-change in parameters (singly or multiply) from a single starting parameter set [26], [41], [42], whereas global analysis tests parameter sets that sample a defined region of parameter space [43]. The approach was supported, for example, in the *Xenopus* cell cycle model, whose plausibility as the relatively more realistic model [44] was later strengthened by also possessing higher robustness [27]. The success of this analysis strategy is further demonstrated by other biological systems [45], [46].

In this study, we investigated the effects of two common complexities, the multiple loop structure and nonlinearity of the kinetics, on model sensitivity and robustness. Firstly, we examined the effect of the multiple loop topology found in many clock models on model sensitivity, using simple modified Goodwin oscillators. We show that multiple loop models have been developed to explain complex behaviours in *Arabidopsis thaliana*, and these models are employed here to test the effects of varying nonlinearity.

## Methods

### Simple oscillator models

**Figure 1:**
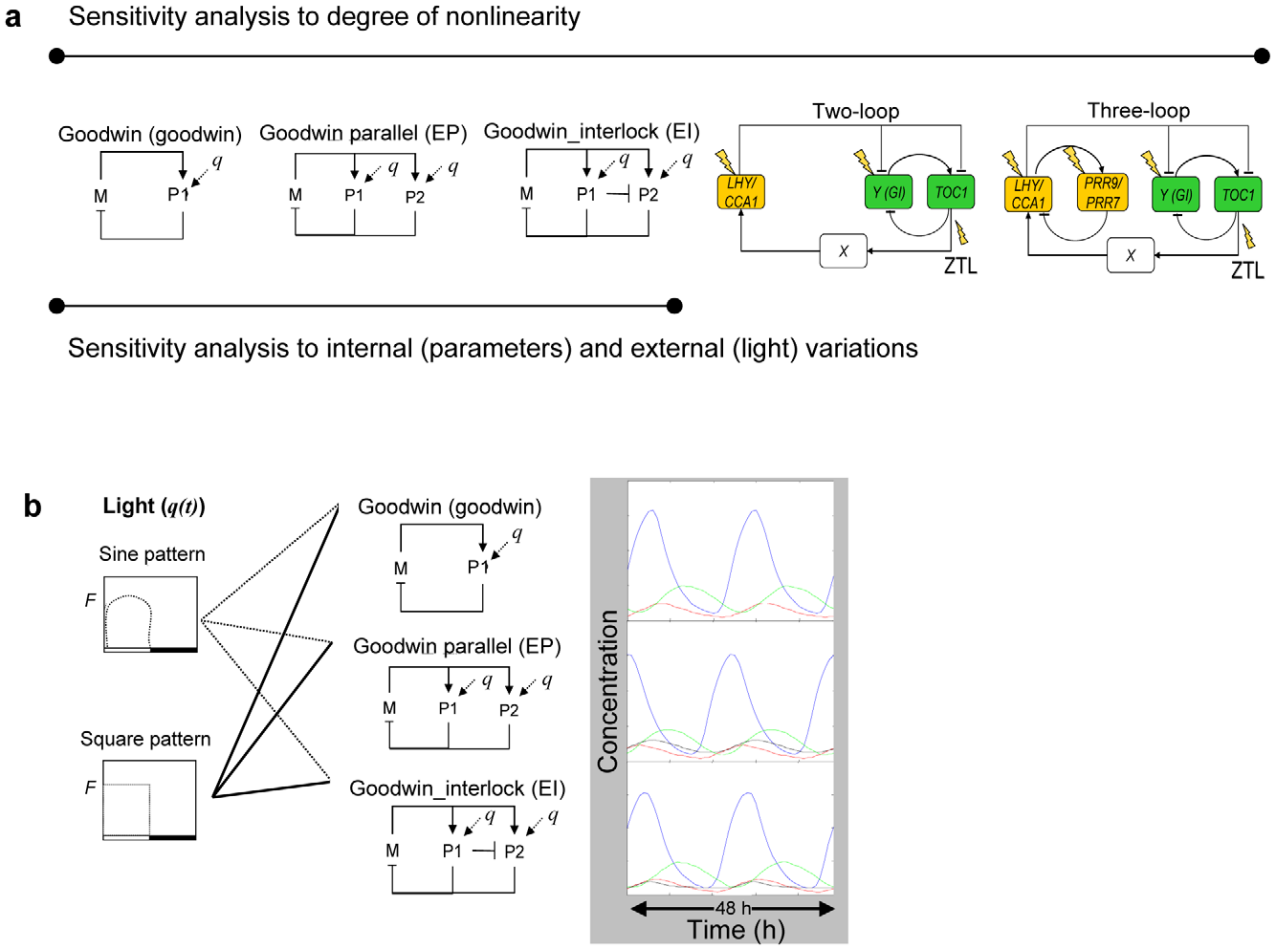
Scheme of models under study and strategic methods. (a) The models in this study consist of three simple Goodwin-type models used for all analyses and two detailed Arabidopsis circadian clock models used in the sensitivity analysis for the degree of nonlinearity. (b) The scheme describes the strategy for investigating the model sensitivity to external variations. The modified Goodwin models were subjected to both light regimes and the subsequent output oscillations were observed and employed to estimate the sensitivity of the model to environmental signal.

In this work, we consider three model topologies of modified Goodwin-type with varying degrees of complexity in the model components and the structure of the circuit. As shown in Figure 1, the modified single-loop Goodwin model (Goodwin; Equations 1a–c) was extended to two multi-loop structures with two forms of the transcriptional repressor, P1 and P2, a parallel-loop Goodwin model (denoted EP; Equations 2a–d) with an additional negative feedback loop parallel to the single loop model, and an interlocking Goodwin model (denoted EI; Equations 2a–c and 3d) which includes an extra interlocking interaction between the multiple loops. The studied oscillators were given comparable mechanisms for sensing an environmental signal through increased synthesis of the repressor(s). The sensitivity and response of the clock to light or other external signals allows the entrainment of the endogenous timer to the surrounding environment.

All observed models were assembled from three molecular components *M*, *E* and *P1* with an additional component *P2* only for EP and EI extended models. The kinetic equations describing the three models are as follows.

For the Goodwin model:
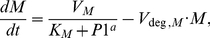
(1a)

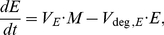
(1b)

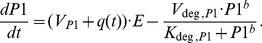
(1c)For the EP model:
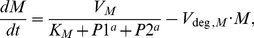
(2a)

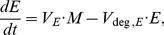
(2b)

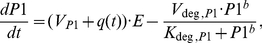
(2c)

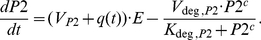
(2d)The EI model is given by Equations 2a–c for *M*, *E*, and *P1*, while the Equation for *P2* is given by:
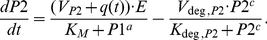
(3d)

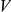
 and 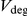 denote the maximal synthesis and degradation rates of model components, 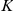 and 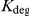 depict kinetic constants of the synthesis and degradation processes, *a*, *b*, and *c* are Hill coefficients and *q(t)* is the light input signal which depends on the time of day. Note that in the Goodwin-type models described above, Hill-type degradation rates have been considered for the repressors so that reasonable amplitude oscillations can be generated for all circuits while remaining within a biologically sensible range for the Hill factor for M transcription (*a*).

The parameters for the studied models were randomly searched through cost optimisation of matching all output profiles (*M*, *E*, and *P*) to a standard sine waveform of 24h-period and unit amplitude to achieve a circadian rhythm with a reasonable size of oscillation. To limit the complexity beyond the scope of study, the EP and EI models were implemented with an identical number of parameters. The parameters were initially searched to optimise the single-loop Goodwin model, which were then fixed during the parameter searching for the extended Goodwin models. Thus, for example, the four additional parameters in the EP extended model (Equation 2d) were varied with the ten parameters in Equations 2a to 2c fixed. The resultant optimal parameter sets of the models are listed in Data S1 in Table 1.

For the Arabidopsis circadian clock models, the parameter sets were given by sequential optimisation strategy against a semi-quantitative (or penalty) cost function [20] and a chi-square cost function [47]. The parameter sets were firstly searched throughout the parameter space, employing the optimisation algorithm established with the one-loop model [20], which tests the phases and periods of simulated clock gene expression profiles. The resulting parameter sets were further refined through simulated annealing optimisation to minimise a chi-square cost of fitting simulations to multiple timeseries data sets that represented a substantially overlapping set of gene expression profiles. The final parameter sets were collected in Data S1 in Tables 5 and 6 for the two-loop and three-loop models, respectively.

### Sensitivity to environment

The three oscillators were tested for their sensitivity to environmental signals. The resulting oscillations were investigated following 10 days of an entraining period to ensure stable oscillations are obtained. We used similar criteria to those of Brandman *et al.* (2005) in which sensitivity is determined by the change in peak time (phase) relative to the reference waveform [44]. The sensitivity of the models to qualitative (patterns of light profile) and quantitative alterations (levels or strengths of light) of the signal was observed in this study through the changes in the output oscillations as illustrated in Figure 1. Sine (Equation 4a) and square (Equation 4b) profiles of light (*q(t)*) were applied to the models during daytime (*q* = 0 after dusk) where *F* is the strength of the light signal (*F*; the amplitude for the sine waveform or the high level of the step function for the square waveform). To examine the sensitivity to signal variation within daytime light, a perturbation, *vs*, to the light signal was introduced in the form of a collective sine function (Equation 4c). This form of variation (*vs*) provides a smoothly changing amplitude wave, modified through variation of *α*, *β*, and *γ* factors, which characterise the perturbation to the light variation signal (a number of factor sets were employed to determine the generality of the results).
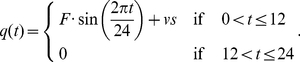
(4a)

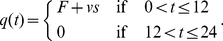
(4b)

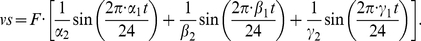
(4c)


### Sensitivity to internal variation (model parameters)

To investigate sensitivity of the model oscillators with respect to parameter variations, each parameter was singly perturbed across a 36-fold range centred on the reference values. The changes to model behaviours were summarised in a single factor called ‘*Degree of Sensitivity*’ (*DOS*; Equation 5), which measures the goodness of fit of simulations to data (or to reference waveform). We denoted by *l* = *1*…*N_m_ ( = 3)* the model, *j* = *1* … *N_p_* the *j^th^* parameter in the parameter set of size *N_p_* and *i* = *−N_a_* …+*N_a_* the *i^th^* perturbation to each parameter, where *N_a_* is the number of positive/negative perturbations and *i* = 0 denotes the unperturbed parameter values (identical to the optimised reference parameters).

We define *C_l,i,j_ (x_e_,x_m_)* to be the *chi-square* cost function [48] calculated at the *i^th^* perturbation to the *j^th^* parameter in the *l^th^* model, where *x_e_* represents an experimental or reference data set to be compared with its counterpart *x_m_* calculated through simulation of the model. The *DOS_l,i,j_* is calculated at each perturbation of each parameter of each model as
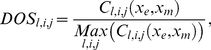
(5)

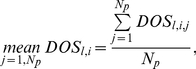
(6)

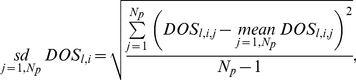
(7)

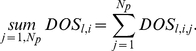
(8)To compare the sensitivity between models we integrate values of *DOS* across the parameters *j* within an individual model by using simple statistics, including arithmetic average (*mean*; Equation 6), standard deviation (*sd*; Equation 7), and summation (*sum*; Equation 8).

### Sensitivity to degree of nonlinearity

The original models of the Arabidopsis clock used non-linear degradation terms for all variables, and the modified Goodwin models include non-linear degradation of the repressors. The number of non-linear terms in the models was reduced through linearisation of *some* or *all* degradation rates, introducing mass action kinetics. To illustrate the procedure, consider the general kinetic equation (Equation 9) and its linearised equivalent (Equation 10). An initial value for *V′* in Equation 10 was determined through the ratio of Michaelis-Menten maximum velocity (*V_max_*) and Michaelis-Menten constant (*K_m_*) in Equation 9. This process yields the “estimated parameter set”. Optimisation was then performed as described above to obtain a new reference parameter set, denoted the “optimised parameter set”. The sensitivities of the fully nonlinear degradation (FND; all degradations follow nonlinear form as shown in Equation 9), partially linear degradation (PLD; some of the degradations follow the linear form as shown in Equation 10) and fully linear degradation (FLD; all degradations follow the linear form) models were compared through the *DOS′* factor (Equation 11) of sensitive parameters.
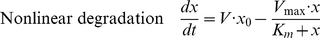
(9)

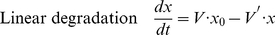
(10)

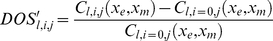
(11)Sensitive parameters for the models are conceptually defined as parameters for which small perturbations lead to a highly deviated profile in the reference waveform. The procedure to classify sensitive parameters follows the consistent robustness analysis method (CRA), described in greater detail in a separate paper [47]. In brief, sensitivity is measured through comparison of the deviated output profile against the reference waveform through the iteration of a single parameter perturbation. The computed results are used to calculated sensitivity coefficients which are the indicators of parameter sensitivity. The group of parameters with high sensitivity coefficient is selected to determine the *DOS′* factor.

## Results

The Goodwin model was originally developed by Goodwin (1965) to understand the generation of spontaneous oscillatory behaviour in an organism [49] and has been widely used as a simple model that can reproduce the physiological behaviour of biochemical oscillators [48]. This simplicity is exploited here to explore the structure of various modified circadian clocks through sensitivity analysis. The Goodwin models were slightly modified (see Methods) and extended to include parallel (EP) and interlocking (EI) loops, which are common structures in complicated circadian models. Parameter values that gave robust oscillation from all models (see Methods) were used to determine the contributions from specific model topologies and the degree of nonlinearity. Based on these results, we extend the investigation to the two-loop and three-loop Arabidopsis circadian clock models [21], [25].

### 1. The significance of multi-loop structure in model sensitivity

A number of studies have revealed that a multi-loop structure increases the capacity for a model to describe complex behaviour in many biological processes [9], [19]–[21], [25], [26], [50], yet it remains to be determined how a particular structure enables/facilitates the desired behaviour.

#### Sensitivity of multi-loop models to internal variations under constant environmental conditions

**Figure 2:**
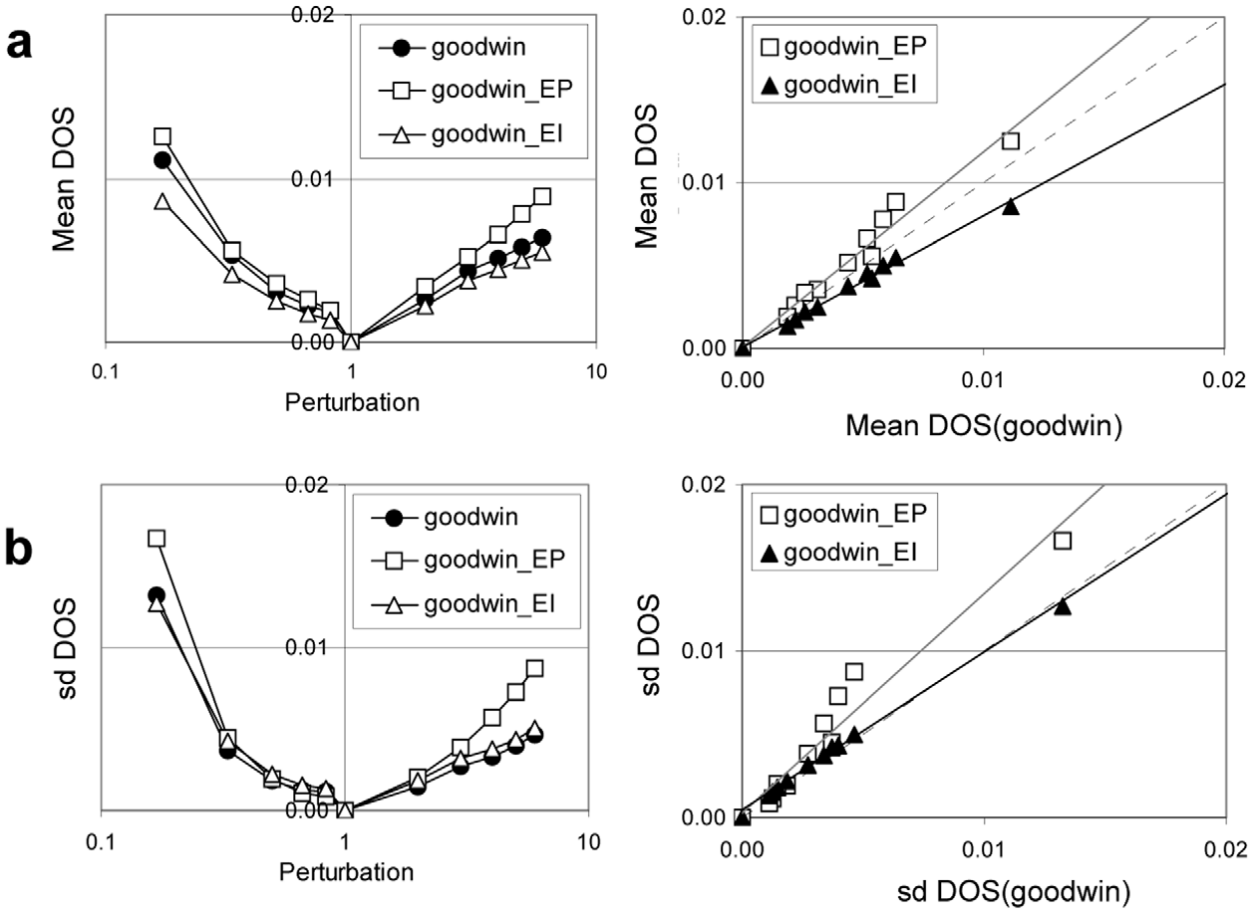
Sensitivity to parameter variations. The sensitivity to parameter variation of modified Goodwin models using parameter set 1 (Data S1: Table 1). The results present statistics of the *DOS* across the model parameters: (a) mean and (b) standard deviation. The calculated *DOS* of models were plotted at each perturbation on the left panel, whereas *DOS* for any perturbations in the multi-loop models were plotted against that of single-loop model on the right panel.

Internal variation (*e.g.* natural mutations) is inevitable in biological systems and, consequently, critical functions should be resistant to such perturbations. As described in the Methods, the sensitivity of a model to internal variation can be investigated through parameter perturbations, measured here by determining the degree of sensitivity (*DOS_l,i,j_*) and their statistics (Equations 6–8). Both the *means* and *sums* indicate the overall sensitivity of the model. Both measurements are helpful for comparing models, yet the *mean* is of greater use for models containing different number of parameters. The s*tandard deviation* provides insight into the variation among individual parameters (which can be considerable). Figure 2 illustrates the sensitivity of the modified Goodwin, EP, and EI models across the full perturbation range represented in terms of *mean DOS_l,i_* and *sd DOS_l,i_*. As seen in Figure 2, the EI model shows the greatest robustness against parameter variations for any statistics of *DOS_l,i_* followed by the Goodwin and the EP models. The robustness of EP and EI models was explicitly compared against the Goodwin model for all perturbations by plotting their *mean DOS_l,i_* and *sd DOS_l,i_* against those of the Goodwin model (Figure 2; right panels). The results clearly indicate that the interlocking model increases the robustness to parameter variation over the simpler Goodwin model, while the parallel model decreases the robustness. Similar results were observed from the same analysis using independent parameter sets, thus indicating the results are not parameter set-specific (Figures S2 and S3). These results indicate that the addition of a loosely-connected loop to the model decreases robustness, yet the robustness can be rescued through inserting appropriate linkage between the loops.

#### Sensitivity of multi-loop models to external variations

**Figure 3:**
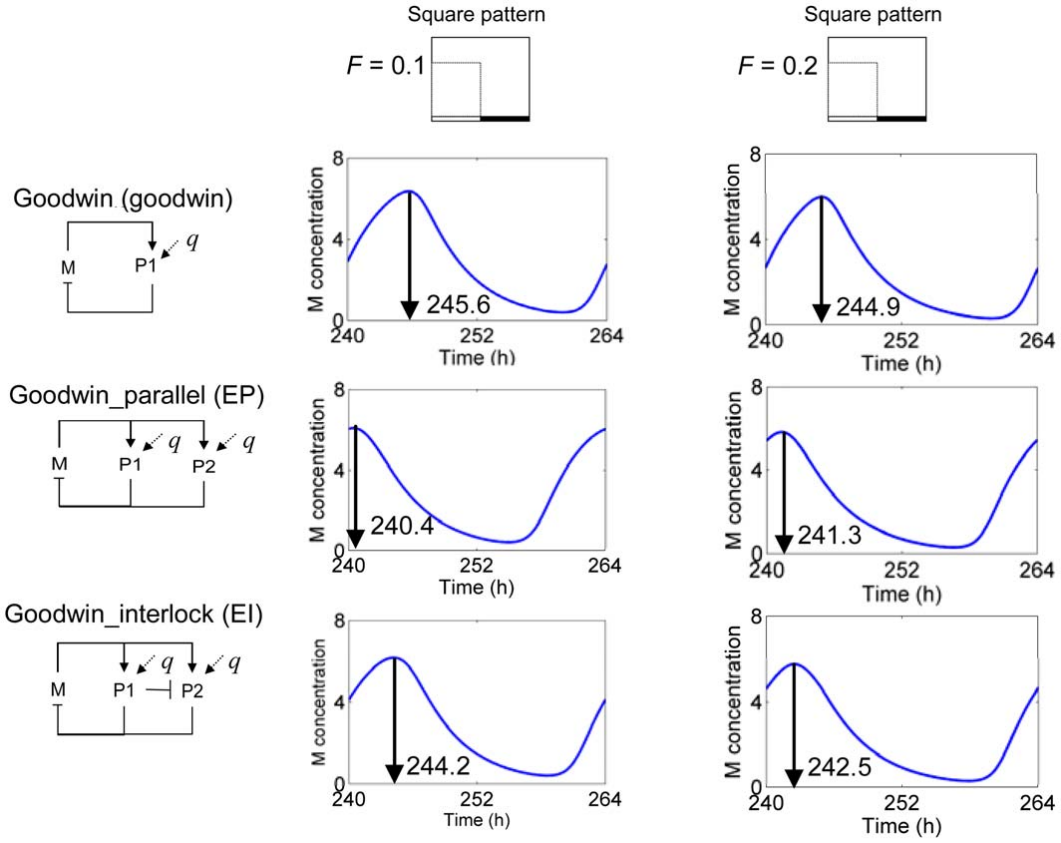
Sensitivity to the strength of external signal. The sensitivity of models to the strength of external signal (*F*) using a square waveform of light *q(t)*.

Circadian systems have evolved to respond to certain environmental changes, *e.g.* sensitivity to the length of the day, yet should be insensitive to other variations, *e.g.* rapid variation in light input across the day. The single-loop Goodwin, EP and EI models were tested to determine their sensitivity to the magnitude of light input using a square waveform. The results plotted in Figure 3 indicate that the EI model displays a greater response to light quantity than the Goodwin and EP models, as suggested from the phase shifts of their output profiles for M: an increase in the light strength *F* from 0.1 to 0.2 results in a 1.7-h phase advance in the peak in M for the EI model while EP and Goodwin model show 0.9-h delayed and 0.7-h advanced peaks respectively. It is noteworthy that the EP model displays an opposite direction phase-shift, implying distinct dynamic transitions in the adjustment of the system.

**Figure 4:**
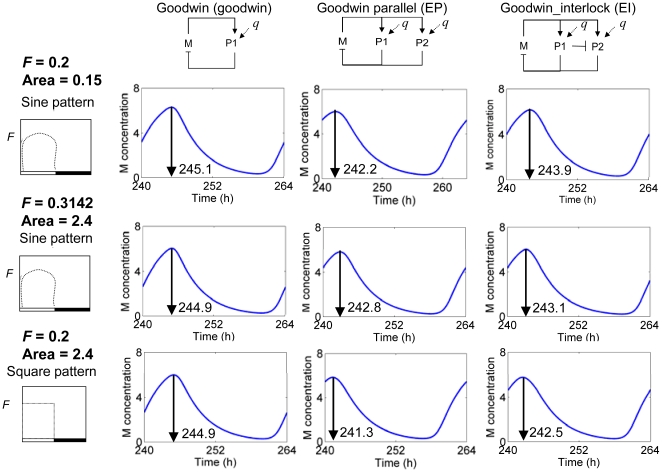
Sensitivity to the pattern of external signal. The sensitivity of the models to the pattern of external signal. The sensitivity to sine- and square-waveform of light having identical strength of signal (*F* = 0.2) and area under curve were compared.

Furthermore, sine and square light waveforms with identical strength (*F* = 0.2) were applied to Goodwin, EP and EI models to examine their ability to distinguish the patterns of light. This experiment was reinforced by an explicit test using an identical quantity of light (*i.e.* equal area under light profiles) as shown in the middle row of Figure 4. The multi-loop models can be distinguished in their response to the different light patterns through their phase-shift (Figure 4), while the single-loop Goodwin model exhibits less change (0 to 0.2-h and 0.6 to 1.5-h shifted in phase for single-loop and multi-loop, respectively). For the light profiles of *F* = 0.2 (top and bottom rows of Figure 4), the EP and EI models with the square waveform produce phase-advanced oscillations relative to that with the sine waveform, which is partially due to greater light input at dawn and dusk. However, the subsequent test for equivalent integrated light profiles (middle and bottom rows of Figure 4) confirmed the given results by showing similar consequences. This double-controlled study (*F* and area under curve) suggests that EP and EI models have the ability to discriminate and respond to the different characteristics of light as well as its quantity.

**Figure 5:**
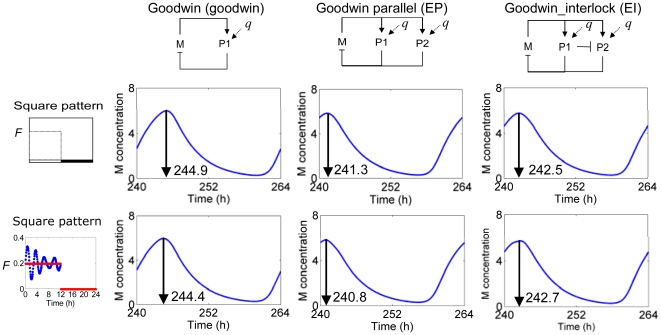
Robustness to the variations of external signal. The robustness of models to variations in the external signal were observed using the square waveform of light with the same strength of signal (*F* = 0.2). Distinct variations were formulated from the collective sine function with different values of variation coefficients to test the generality of the results, with the plotted data representative of all such applied variations. The results plotted were obtained under the following variation coefficients: *α1* = 8, *α2* = 3, *β1* = 9, *β2* = 5, *γ1* = 10 and *γ2* = 7.

Resistance to external fluctuations is also an important feature for circadian clocks, and we therefore tested the model robustness to a square light waveform with continuous variation (Equation 4c) superimposed. According to the phase-shift and characteristics of the output profiles, all models display an ability to resist such variations (Figure 5). Nevertheless, the EI model seems to show greater robustness, maintaining its peak-time within a 0.2-h deviation, over the EP and Goodwin models (phase shift of 0.5 h). Similar results were obtained with other variations, formulated through altering the characteristic factors *α*, *β* and *γ* in Equation 4c. In summary, the results suggest that a multiple negative feedback loop structure confers desirable properties through enhancing sensitivity to both qualitative and quantitative changes of the photo-profiles and their robustness to noisy external fluctuations.

### 2. The effect of nonlinear kinetics on model sensitivity

#### The effect of nonlinear kinetics on the sensitivity of modified Goodwin models

**Figure 6:**
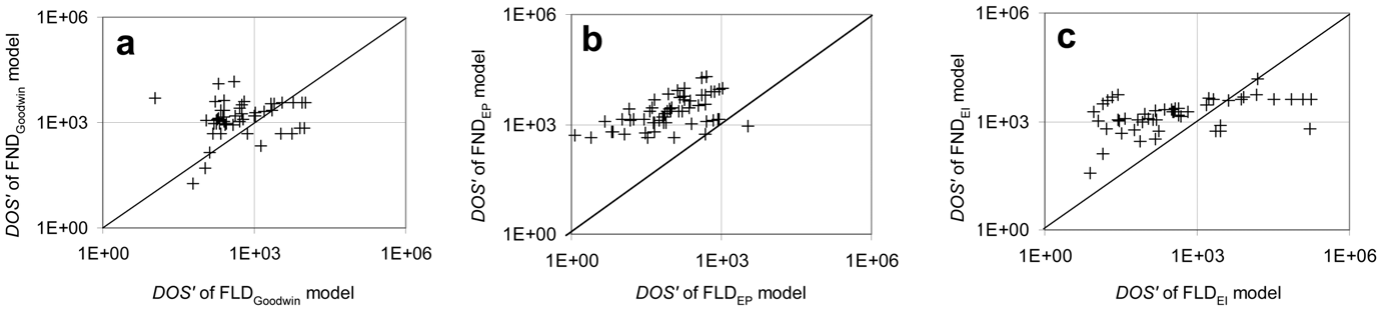
Sensitivity to the nonlinearity of modified Goodwin models. The sensitivity of the Goodwin-type models with varied degree of nonlinearity is presented by plotting the *DOS* of sensitive parameters from partially linearised model (PLD) against its corresponding fully nonlinear model (FND): (a) single-loop Goodwin model (b) Goodwin parallel (EP) (c) Goodwin interlocking (EI).

Difficulties in measurement hinder experimental identification of the interactions occurring in a system. Michaelis-Menten kinetics are often used *ad hoc* to model biochemical reactions that are expected to saturate, yet their employment introduces an additional nonlinearity which may or may not be necessary. The original models of the Arabidopsis clock used non-linear degradation terms for all variables, for example, and the modified Goodwin models include one non-linear degradation term. To determine the impact of this nonlinearity, the sensitivity of the Goodwin models (single-loop, EP, and EI) was compared, with the original nonlinear degradation term (the model form denoted Partially Linear Degradation, PLD) or after converting this term to a linear degradation term (Fully Linear Degradation, FLD; the linearised model equations are presented in supplementary material). Each point (+) in Figure 6 represents the *DOS* of sensitive parameters from more or less nonlinear models over all perturbations. The sensitive parameters were classified as described in Methods. The diagonal line is the iso-sensitivity line, indicating identical sensitivity between two compared models. The results indicate that for all models a greater degree of nonlinearity leads to higher sensitivity. The modified Goodwin models contain a relatively low degree of nonlinearity compared to real system models, and we therefore extended this analysis to test if the same results are obtained from more complicated models.

#### The effect of nonlinear kinetics on the sensitivity of Arabidopsis circadian clock models

Both the two-loop [21] and three-loop models [25] for the Arabidopsis circadian clock employ Michaelis-Menten terms for all degradation kinetics (this form of the model is denoted here as Fully Nonlinear Degradation, FND). To investigate the impact of nonlinear kinetics on model sensitivity, both models were partially linearised (denoted here as Partially Linear Degradation models, PLD) by replacing the Michaelis-Menten degradation terms with mass action kinetics as described in Methods. The linearisation was performed stepwise for each degradation term and repeated until good-fit simulations did not persist following re-optimisation of the model to the same sets of data, *e.g.* due to loss of oscillation. This is to ensure equivalent performance between the various models. The sensitivity to parameter variations was investigated for a variety of two-loop and three-loop models and the results were analysed through comparison of *DOS′* of the sensitive parameters, selected based on CRA.

#### Partial linearisation of model kinetics

Linearisation of the degradation rates not only reduces the degree of nonlinearity in the model but also the number of parameters needed to simulate the model. Ten parameters were eliminated from the two-loop model (FND_2loop_) [21] without significantly altering its capacity to fit to data or desirable experimental behaviour (*C_l,i,j_(x_e_,x_m_)* changes from 1.12 to 0.49, representing a better fit to the data). All *RNA* and protein degradation in the linearised model (PLD_2loop_) follows mass action kinetics with the exception of nuclear and cytoplasmic TOC1 proteins. Figure S4 depicts the equivalent goodness of fit between model simulations and data of the PLD_2loop_ and FND_2loop_ models. The only significant difference between the two models is the eight-fold decrease in acute light induction of *Y/GI* in the PLD_2loop_ model. The two-loop partially linear degradation model and its re-optimised parameters are listed in Data S1 in Table 5.

Due to the extremely high sensitivity of parameters reported in the original three-loop model [25], a global optimisation of the model was initially performed to aid further model modifications. The resulting parameters are shown in Data S1 in Table 6. Linearisation of the three-loop model (FND_3loop_) enabled a reduction of six parameters from 74 without significant loss in its capacity to fit the data (1.16 and 1.12 for nonlinear and linear models; Figure S5). *LHY* and *PRR9/PRR7* mRNAs and proteins degradation were all modelled with linear forms in the modified three-loop model (PLD_3loop_) while the remaining (*TOC1*, *X*, and *Y* mRNAs and proteins) required Michaelis-Menten degradation kinetics. The reduced ability to linearise the three-loop in comparison to the two-loop model indicates that the three-loop model requires greater complexity to satisfy the additional data sets involving *PRR9*, *PRR7* and the *prr9prr7* double mutant. Although the PLD_3loop_ model gave similar results of data fitting, it again loses some ability to respond to light as indicated in the *Y/GI* simulation. The consistency in this reduced capacity for both partially linear degradation models implies that retaining the nonlinearity in the *Y/GI* component is crucial for fully capturing plant behaviours previously simulated by the nonlinear degradation models. Possibly, a saturating rate of degradation for a certain molecular entity in the models is necessary in generating time-delayed oscillations [51].

#### Sensitivity of Arabidopsis circadian clock models with varying degrees of nonlinearity

The sensitivities of the nonlinear and partially linearised models were qualitatively and quantitatively compared for both two-loop and three-loop models. The sensitive parameters from each model were determined from the consistent robustness analysis as listed in Table 1 (for further detail see [47]). Nine sensitive parameters were identified from the 58 parameters in the FND_2loop_ model (mainly involving *TOC1* transcription), while another nine sensitive parameters were identified from 48 parameters of the PLD_2loop_ model. Noteworthy is the large intersection between the two versions of the two-loop models, suggesting that linearisation did not significantly alter the dynamics of the model. For the three-loop model, the FND_3loop_ model yielded 14 sensitive parameters from 74, relating to *TOC1* and *Y* transcription and degradation, while the corresponding PLD_3loop_ model demonstrated a highly overlapping set of ten sensitive parameters from 68. The conservation between sensitive parameters in the nonlinear degradation and partially linearised three-loop models suggests again that *selective* linearisation did not substantially affect model dynamics.

**Table 1:** Summary of the sensitivity analysis for two-loop and three-loop models.

Descriptions	Two-loop model		Three-loop model	
	FND_2loop_	PLD_2loop_	FND_3loop_	PLD_3loop_
Number of sensitivity parameters	9	9	14	10
Sensitive parameters	n2		n2	
	g3	g3	n3	
	m4	m4	n4	n4
		m9	n5	
	k4	k4	g2	g2
	k7		m4	m4
	p2		m9	
	p3	p3	m12	m12
		r5	m13	m13
		a		
	b	b	k10	k10
	d	d	p4	p4
				r8
			b	b
			e	
			f	
				g
Percent of intersection	(6/9)×100 = 66.7%		(8/10)×100 = 80%	

**Figure 7:**
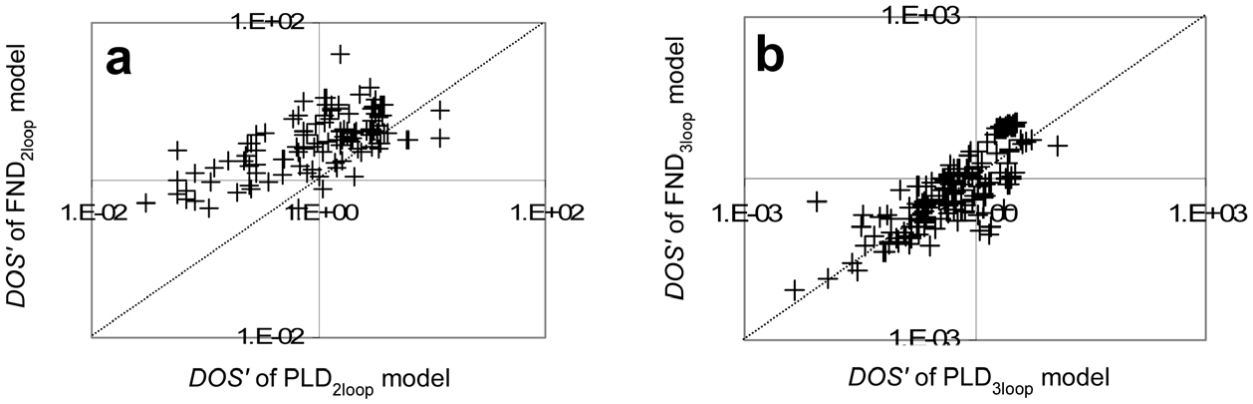
Sensitivity to the nonlinearity of Arabidopsis circadian clock models. The sensitivity of varied degree in nonlinearity of Arabidopsis circadian clock models is presented by plotting *DOS* of sensitive parameters from the partially linearised model (PLD) against its corresponding fully nonlinear model (FND): (a) two-loop model (b) three-loop model.

The sensitivities of the partially linearised models were compared quantitatively with their fully nonlinear counterparts using the sensitive parameters as depicted in Figure 7. Each point (+) represents the *DOS′* of sensitive parameters for each model across all perturbations. The results show that for both the two-loop and three-loop models the *DOS* points generally lie above the iso-sensitivity line (especially for the most sensitive parameters). Consistent with the results for the modified Goodwin models, sensitivity to parameter variation was lower in the partially linearised models than the corresponding nonlinear degradation models.

## Discussion

Complexity is an inevitable consequence of iterative extension of models to simulate new data, with multi-loop structures and nonlinearity common examples of such complexities. In the specific example of the Arabidopsis circadian clock, a sequence of models has been published with an increasing degree of complexity from one-loop to four-loop [20], [21], [25], [26]. While certain complexities are an intentional and necessary inclusion to extend the boundaries of the model and its capability to include known network topology, unnecessary complexity may arise from inclusion of features that are possible but not demonstrated, such as the saturation of cellular degradation pathways or the independent functions of parallel regulation. This complexity potentially hinders the ability to apply mathematical analyses and it is appropriate to question whether all complexities are required.

Increased complexity affects both the behaviours and properties of the model. Besides the remarkable behaviour in producing rhythms with accurate phase and period, the circadian clocks in diverse organisms balance properties of sensitivity and robustness: the circadian clock is not only robust to fluctuating signals (external noise) and intrinsic variations [33], [40], but it is also sensitive to certain environmental clues such as the daily light-dark cycle [31], [52], [53]. Models of the circadian clock are therefore expected to be sensitive to different kinds of effectors. As sensitivity is a key property for inferring the plausibility of the model, sensitivity analysis has been used to explore the significance of the model's complexities regarding multi-loop structure and nonlinearity.

Multiple negative feedback loop structures are common in circadian clock models ranging from *Synechococcus* cyanobacteria [54] to plants [21], [25] and mammals [19]. Single loop structures are incapable of describing the properties of circadian clocks in living organisms [9], [20], [50] and ignore molecular evidence for multiple loop connectivity. The results in Figures 2, 3, 4, 5 show that the multi-loop structure can affect the sensitivity of the systems, balancing sensitivity to external clues with robustness against internal variation. Compared with the simple single loop structure, the multiple loop circuits enhance the ability to sense and respond to the amplitude (Figure 3) and waveform (Figure 4) of input signals while maintaining high robustness against noisy input (Figure 5). Surprisingly, relative to the single loop model, the two related circuits of multi-loop structure (EP and EI) demonstrate opposite sensitivity to internal parameter variation (Figure 2). According to the detailed sensitivity analysis (CRA), the high sensitivity in the EP model reflects the non-uniform sensitivity of the model with respect to parameters, arising from the parallel connections introduced to the EP network. The former is reflected by the higher *sd DOS_l,i_* of the EP to those of EI models, as the sensitivity of the EP model derived from a single highly sensitive parameter. The latter is interpreted from the sensitive parameters identified for each model. Parameters involving regulation from *P1* and *P2* to *M* were less significant in the EI than EP models, due to the additional inhibition of *P1* to *P2* in the EI model. In conclusion, the high sensitivity of the EP model relative to the modified Goodwin model may arise from an inappropriate incorporation of a new loop structure, since this sensitivity was vastly improved by the extra incorporation of a single interlocking connection into the model, EI. This may suggest that a compact, multiply-connected architecture is also necessary for increasing the robustness of the model against internal variations [28], [55]–[57]. Considering all of the above parts, the interlocked negative feedback loop model (EI) shows many advantages as a common structure for circadian clock circuits.

Nonlinearity in the kinetics is another complexity often introduced through modelling. Nonlinear kinetics are required to produce oscillations, particularly in small models, and the necessity for nonlinearity decreases with increased size (or other complexities) of the model. Here we show that nonlinearities can increase the sensitivity of model behaviours to parameter perturbation, even in simple models (Figure 6). Similarly, many of the nonlinear degradation terms of previous models were not necessary for improving the fit to Arabidopsis circadian clock data. Comparable fits were obtained from models with varying degrees of nonlinearity (Figures S4 and S5). Furthermore, the excessive nonlinearity introduced additional sensitivity to the models: the qualitative (Table 1) and quantitative (Figure 7) comparisons suggest that nonlinearity also increases the sensitivity of these complex models, without major perturbation to the dynamics of the model. The sensitive parameters were conserved between the nonlinear (FND) and partially linearised (PLD) models despite having different degradation kinetics (Table 1). Understanding the role of the nonlinearity in the model enables us to minimise unnecessary model complexity, which decreases both the sensitivity of the model and the number of parameters. An exploration test on the computational time for models differing in the dimension of parameter space resulting from partial linearisation (Figure 6S) exemplifies the advantage of the less complex models for further mathematical analyses.

The most valuable model is the simplest model that retains enough adaptability to explain the real system behaviour and also matches the system's properties. Besides these properties, models with strong predictive capacity and heuristic value are the ultimate goal of modelling. The study of model structure and its kinetics allows us to understand the contributions of complexities in molecular networks to the behaviours and intrinsic properties of the model. Applying this understanding, we can effectively reduce the complexity of a model while its adaptability is retained, so it may become an even more useful and predictive tool.

### Conclusions

Circadian clocks are one of the biological systems that have been modelled extensively to gain more comprehension about regulatory mechanisms. In this work, we showed that the complexity from multi-loop structures may be required to improve the behaviours and properties of the models, whereas excessive nonlinearity weakens the robustness of the models. In particular we identified the interlocking loop structure as providing a suitable model architecture for the circadian clock and other robust biochemical oscillators, because this circuit provides a good tradeoff in sensitivity and robustness to input signals and noisy input. We also found that the degree of nonlinearity in two models of the Arabidopsis circadian clock, the two-loop and three-loop models, can be diminished and still produce more robust models with equivalent model behaviours. The multi-loop structure and nonlinearity are indeed only two of the abundant complexities found in the models, but they are commonly used. In brief, this work will enable the effective extension of mathematical models to include more biochemical components with clear understanding of their impact. Balancing realism against the complexity of the model may promote simpler models, which are beneficial for many subsequent analyses.

## Supporting Information

Figure S1 Model topology of Arabidopsis circadian clock: (a) one-loop (Locke et al., 2005a), (b) two-loop (Locke et al., 2005b), (c) three-loop (Locke et al., 2006), and (d) four-loop models (Zeilinger et al., 2006).
(0.68 MB TIF)

Figure S2 The sensitivity to parameter variation of modified Goodwin models using parameter set 3 (Data S1, Table 1). The results present in term of statistics of DOS across the model parameters: (a) mean and (b) standard deviation. The calculated DOS of models were plotted for each perturbation on the left panel with DOS entities at any perturbations of multi-loop models plotted against that of the single-loop model on the right panel.
(2.19 MB TIF)

Figure S3 The sensitivity to parameter variation of modified Goodwin models using parameter set 4 (Data S1, Table 1). The results present in term of statistics of DOS across the model parameters: (a) mean and (b) standard deviation. The calculated DOS of models were plotted at each perturbation on the left panel with DOS entities at any perturbations of multi-loop models were plotted against that of the single-loop model on the right panel.
(2.04 MB TIF)

Figure S4 Plots showing the best fit of the two-loop Arabidopsis circadian clock model for (a) fully-nonlinear degradation, (b) partially-linearised degradation with estimated parameter set (the initialising parameter set for parameter searching is determined by the ratio of V_max_ and K_m_ in the counterpart nonlinear terms of the fully-nonlinear degradation models), and (c) partially-linearised degradation with optimised parameter set.
(1.06 MB TIF)

Figure S5 Plots showing the best fit of the three-loop Arabidopsis circadian clock model for (a) fully-nonlinear degradation, (b) partially-linearised degradation with estimated parameter set (the initialising parameter set for parameter searching is determined by the ratio of V_max_ and K_m_ in the counterpart nonlinear terms of the fully-nonlinear degradation models), and (c) partially-linearised degradation with optimised parameter set.
(1.18 MB TIF)

Figure S6 The computational cost of using complex models. The running time for 100 annealing steps (numbers of search cycles through a simulated annealing algorithm) for the nonlinear and partially linearised two-loop Arabidopsis circadian clock models: (1) fully nonlinear model - 58 parameters (2) partially linearised RNA degradation - 54 parameters (3) partially linearised RNA and LHY protein degradation - 52 parameters (4) partially linearised RNA, LHY and X protein degradation - 50 parameters and (5) partially linearised RNA, LHY, X and Y protein degradation - 48 parameters. The number of parameters in models is reduced with increasing degree of linearity, resulting in a reduction of the computational time for the optimisation. Computational experiments were performed on a standard desktop computer with Intel^(R)^ Pentium^(R)^ D CPU 3.00GHz 2.99GHz, 1.99 GB of RAM and Microsoft Windows XP Professional Version 2002 operating system.
(1.72 MB TIF)

Data S1 The supplementary data consists of model equations and the corresponding sets of parameter used in our analyses.
(0.54 MB DOC)

## References

[1] Dunlap JC (1999). Molecular bases for circadian clocks.. Cell.

[2] Harmer SL, Panda S, Kay SA (2001). Molecular bases of circadian rhythms.. Annu Rev Cell Dev Biol.

[3] Johnson CH, Golden SS (1999). Circadian programs in cyanobacteria: adaptiveness and mechanism.. Annu Rev Microbiol.

[4] Kondo T (2007). A cyanobacterial circadian clock based on the Kai oscillator.. Cold Spring Harb Symp Quant Biol.

[5] Dunlap JC, Loros JJ (2004). The *Neurospora* circadian system.. J Biol Rhythms.

[6] Brunner M, Kaldi K (2008). Interlocked feedback loops of the circadian clock of *Neurospora crassa*.. Mol Microbiol.

[7] Heintzen C, Liu Y (2007). The *Neurospora crassa* circadian clock.. Adv Genet.

[8] Glossop NRJ, Lyons LC, Hardin PE (1999). Interlocked feedback loops within the *Drosophila* circadian oscillator.. Science.

[9] Ueda HR, Hagiwara M, Kitano H (2001). Robust oscillations within the interlocked feedback model of *Drosophila* circadian rhythm.. J Theor Biol.

[10] Boothroyd CE, Young MW (2008). The in(put)s and out(put)s of the Drosophila circadian clock.. Ann N Y Acad Sci.

[11] Dubruille R, Emery P (2008). A plastic clock: how circadian rhythms respond to environmental cues in *Drosophila*.. Mol Neurobiol.

[12] Alabadí D, Oyama T, Yanovsky MJ, Harmon FJ, Más P (2001). Reciprocal regulation between *TOC1* and *LHY/CCA1* within the *Arabidopsis* circadian clock.. Science.

[13] McClung CR (2006). Plant circadian rhythms.. Plant Cell.

[14] Mas P (2008). Circadian clock function in Arabidopsis thaliana: time beyond transcription.. Trends Cell Biol.

[15] McClung CR (2008). Comes a time.. Curr Opin Plant Biol.

[16] Ko CH, Takahashi JS (2006). Molecular components of the mammalian circadian clock.. Hum Mol Genet.

[17] Hastings MH, Maywood ES, Reddy AB (2008). Two decades of circadian time.. J Neuroendocrinol.

[18] Leloup JC, Goldbeter A (2008). Modeling the circadian clock: from molecular mechanism to physiological disorders.. Bioessays.

[19] Forger DB, Peskin CS (2003). A detailed predictive model of the mammalian circadian clock.. Proc Natl Acad Sci U S A.

[20] Locke JCW, Millar AJ, Turner MS (2005). Modelling genetic networkswith noisy and varied experimental data: the circadian clock in *Arabidopsis thaliana*.. J Theor Biol.

[21] Locke JCW, Southern MM, Kozma-Bognar L, Hibberd V, Brown PE (2005). Extension of a genetic network model by iterative experimentation and mathematical analysis.. Mol Syst Biol.

[22] Farre EM HS, Harmon FG, Yanovsky MJ, Kay SA (2005). Overlapping and distinct roles of PRR7 and PRR9 in the Arabidopsis circadian clock.. Current Biology.

[23] Mizuno T, Nakamichi N (2005). *Pseudo*-Response Regulators (PRRs) or T*rue* Oscillator Components (TOCs).. Plant Cell Physiol.

[24] Nakamichi N, Kita M, Ito S, Sato E, Yamashino T (2005). The Arabidopsis *Pseudo-Response Regulators*, *PRR5* and *PRR7*, coordinately play essential roles for circadian clock function.. Plant Cell Physiol.

[25] Locke JCW, Kozma-Bognar L, Gould PD, Feher B, Kevei E (2006). Experimental validation of a predicted feedback loop in the multi-oscillator clock of *Arabidopsis thaliana*.. Mol Syst Biol.

[26] Zeilinger NM, Farre EM, Taylor RS, Kay AS, Doyle FJ (2006). A novel computational model of the circadian clock in *Arabidopsis* that incoperates *PRR7* and *PRR9*.. Mol Syst Biol.

[27] Morohashi M, Winn AE, Borisuk MT, Bolouri H, Doyle J (2002). Robustness as a measure of plausibility in models of biochemical networks.. J theor Biol.

[28] Wagner A (2005). Circuit topology and the evolution of robustness in two-gene circadian oscillators.. Proc Natl Acad Sci U S A.

[29] Kurosawa G, Mochizuki A, Iwasa Y (2002). Comparative study of circadian clock models, in search of processes promoting oscillation.. J Theor Biol.

[30] Rand DA, Shulgin BV, Salazar D, Millar AJ (2004). Design principles underlying circadian clocks.. J R Soc Interface.

[31] Kim J, Bae W, Yoon Y, Cho K (2007). Topological difference of core regulatory networks induces different entrainmnet charateristics of plant and animal circadian clocks.. Biophysical Journal-Biophysical Letter.

[32] Lindenschmidt K (2006). The effect of complexity on parameter sensitivity and model uncertainty in river water quality modelling.. Ecol Modell.

[33] Kitano H (2004). Biological robustness.. Nature Review Genetics.

[34] Kitano H (2007). Toward a theory of biological robustness.. Mol Syst Biol.

[35] Stelling J, Sauer U, Szallasi Z, Doyle FJ, Doyle J (2004). Robustness of cellular functions.. Cell.

[36] Doyle FJ, Gunawan R, Bagheri N, Mirsky H, To TL (2006). Circadian rhythm: a natural, robust, multi-scale control system.. Computers and Chemical Engineering.

[37] Stelling J, Gilles ED, Doyle FJ (2004). Robustness properties of circadian clock architectures.. Proc Natl Acad Sci U S A.

[38] Zak DD, Stelling J, Doyle FJ (2005). Sensitivity analysis of oscillatory (bio)chemical systems.. Computers and Chemical Engineering.

[39] Barkai N, Leibler S (2000). circadian clocks limited by noise.. Nature.

[40] Wolf J, Becker-Weimann S, Heinrich R (2005). Analysing the robustness of cellular rhythms.. Syst Biol.

[41] Fell DA (1992). Metabolic control analysis: a survey of its theoretical and experimental development.. Biochem J.

[42] Ferreira JS, Lozano R, Mondié S, Friboulet A (2006). Bifurcation analysis of a biochemical network.. Positive Systems.

[43] Kurata H, Tanaka T, Ohnishi F (2007). Mathematical identification of critical reactions in the interlocked feedback model.. PLoS ONE.

[44] Brandman O, Ferrell JE, Li R, Meyer T (2005). Interlinked fast and slow positive feedback loops drive reliable cell decisions.. Science.

[45] Buceta J, Herranz H, Canela-Xandri O, Reigada R, Sagues F (2007). Robustness and stability of the gene regulatory network involved in DV boundary formation in the Drosophila wing.. PLoS ONE.

[46] Masuda N, Amari S (2008). A computational study of synaptic mechanisms of partial memory transfer in cerebellar vestibulo-ocular-reflex learning.. J Comput Neurosci.

[47] Saithong T, Painter KJ, Millar AJ (2010). Consistent robustness analysis (CRA) identifies biologically relevant properties of regulatory network models..

[48] Murray JD (1993). Mathematical Biology.

[49] Goodwin BC, Weber G (1965). Oscillatory behavior in enzymatic control processes.. Advances in Enzyme Regulation.

[50] Leloup J, Gonze D, Goldbeter A (1999). Limit cycle models for circadian rhythms based on transcriptional regulation in *Drosophila* and *Neurospora*.. J Biol Rhythms.

[51] Kurosawa G, Iwasa Y (2002). Saturation of enzyme kinetics in circadian clock models.. J Biol Rhythms.

[52] Eckardt NA (2005). Temperature entrainment of the Arabidopsis circadian clock.. Plant Cell.

[53] Zeng H, Qian Z, Myers MP, Rosbash M (1996). A light-entrainment mechanism for the *Drosophila* circadian clock.. Nature.

[54] Miyoshi F, Nakayama Y, Kaizu K, Iwasaki H, Tomita M (2007). A mathematical model for the Kai-protein–based chemical oscillator and clock gene expression rhythms in Cyanobacteria.. J Biol Rhythms.

[55] Paladugu SR, Chickarmane V, Deckard A, Frumkin JP, McCormack M (2006). *In silico* evolution of functional modules in biochemical networks.. IEE Proc-Syst Biol.

[56] Ciliberti S, Martin OC, Wagner A (2007). Robustness can evolve gradually in complex regulatory gene networks with varying topology.. PLoS Comput Biol.

[57] Kwon Y, Cho K (2007). Analysis of feedback loops and robustness in network evolution based on Boolean models.. BMC Bioinformatics.

