# Supplementary figures and images for: The Contributions of Interlocking Loops and Extensive Nonlinearity to the Properties of Circadian Clock Models
Source: PLoS One. 2010 Nov 30;5(11):e13867. doi: 10.1371/journal.pone.0013867 (PMC2994703; doi:10.1371/journal.pone.0013867)

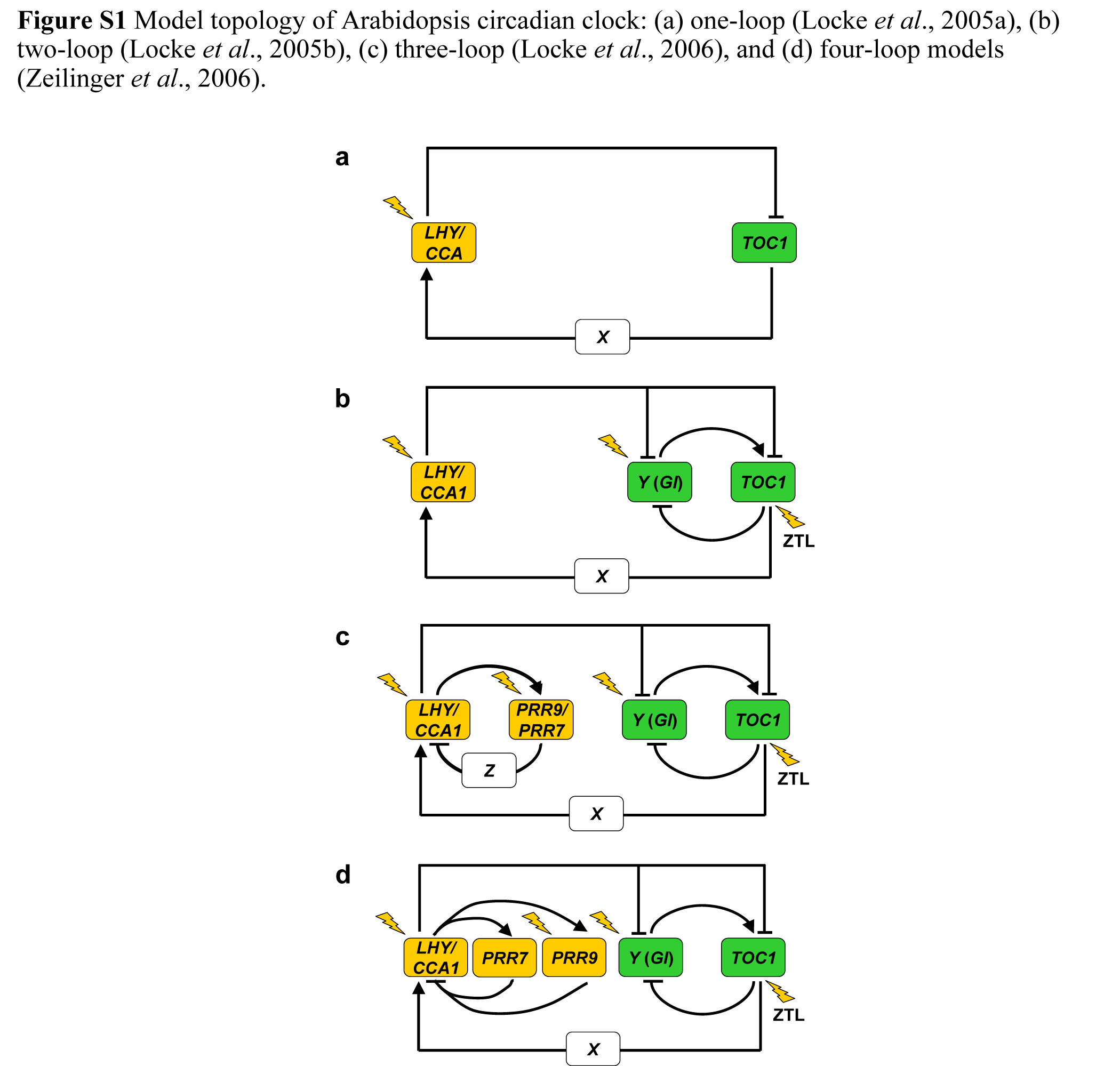

Supplement: Figure S1 — Model topology of Arabidopsis circadian clock: (a) one-loop (Locke et al., 2005a), (b) two-loop (Locke et al., 2005b), (c) three-loop (Locke et al., 2006), and (d) four-loop models (Zeilinger et al., 2006). (0.68 MB TIF) [file pone.0013867.s001.tif]

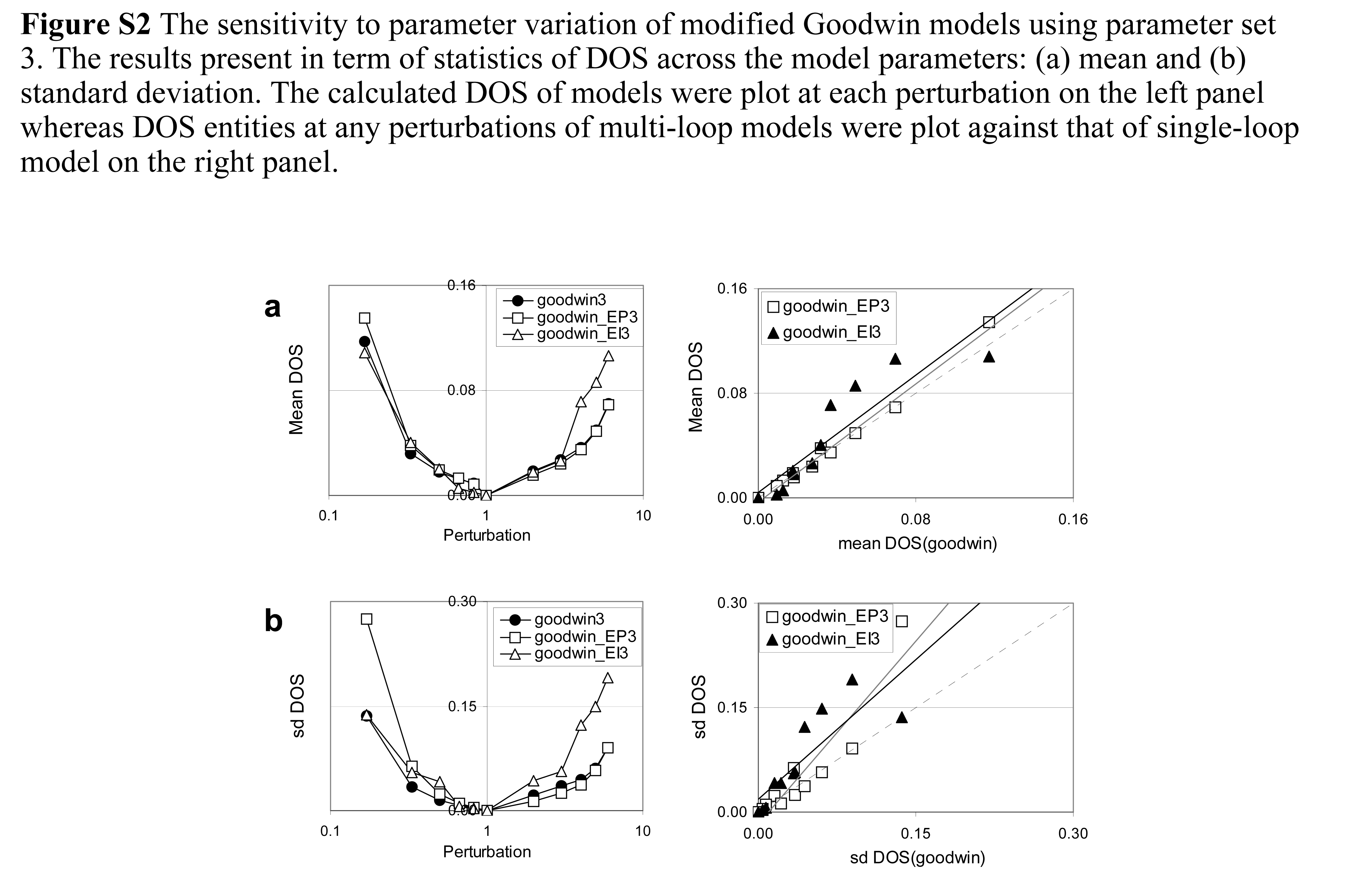

Supplement: Figure S2 — The sensitivity to parameter variation of modified Goodwin models using parameter set 3 (Data S1, Table 1). The results present in term of statistics of DOS across the model parameters: (a) mean and (b) standard deviation. The calculated DOS of models were plotted for each perturbation on the left panel with DOS entities at any perturbations of multi-loop models plotted against that of the single-loop model on the right panel. (2.19 MB TIF) [file pone.0013867.s002.tif]

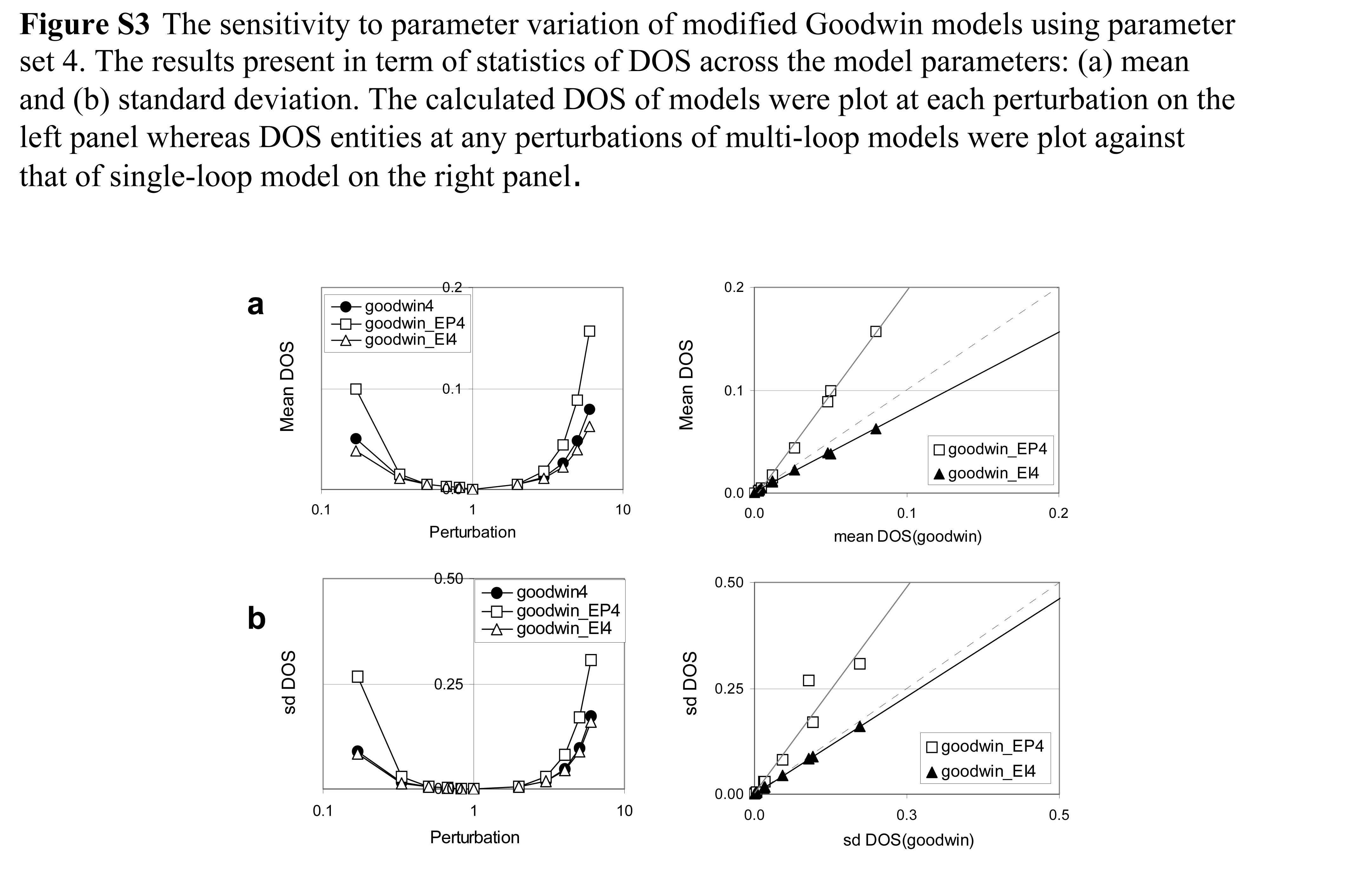

Supplement: Figure S3 — The sensitivity to parameter variation of modified Goodwin models using parameter set 4 (Data S1, Table 1). The results present in term of statistics of DOS across the model parameters: (a) mean and (b) standard deviation. The calculated DOS of models were plotted at each perturbation on the left panel with DOS entities at any perturbations of multi-loop models were plotted against that of the single-loop model on the right panel. (2.04 MB TIF) [file pone.0013867.s003.tif]

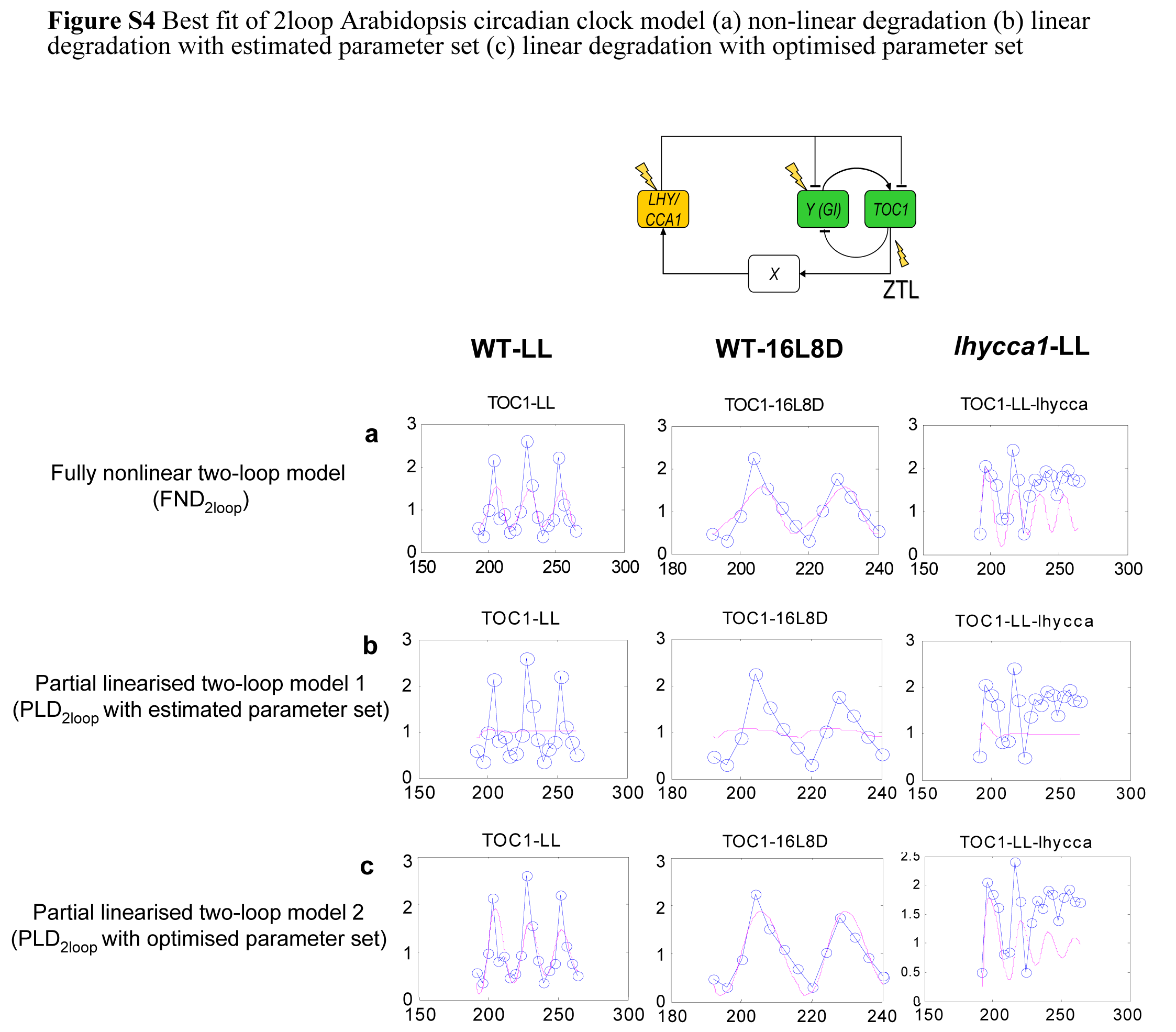

Supplement: Figure S4 — Plots showing the best fit of the two-loop Arabidopsis circadian clock model for (a) fully-nonlinear degradation, (b) partially-linearised degradation with estimated parameter set (the initialising parameter set for parameter searching is determined by the ratio of Vmax and Km in the counterpart nonlinear terms of the fully-nonlinear degradation models), and (c) partially-linearised degradation with optimised parameter set. (1.06 MB TIF) [file pone.0013867.s004.tif]

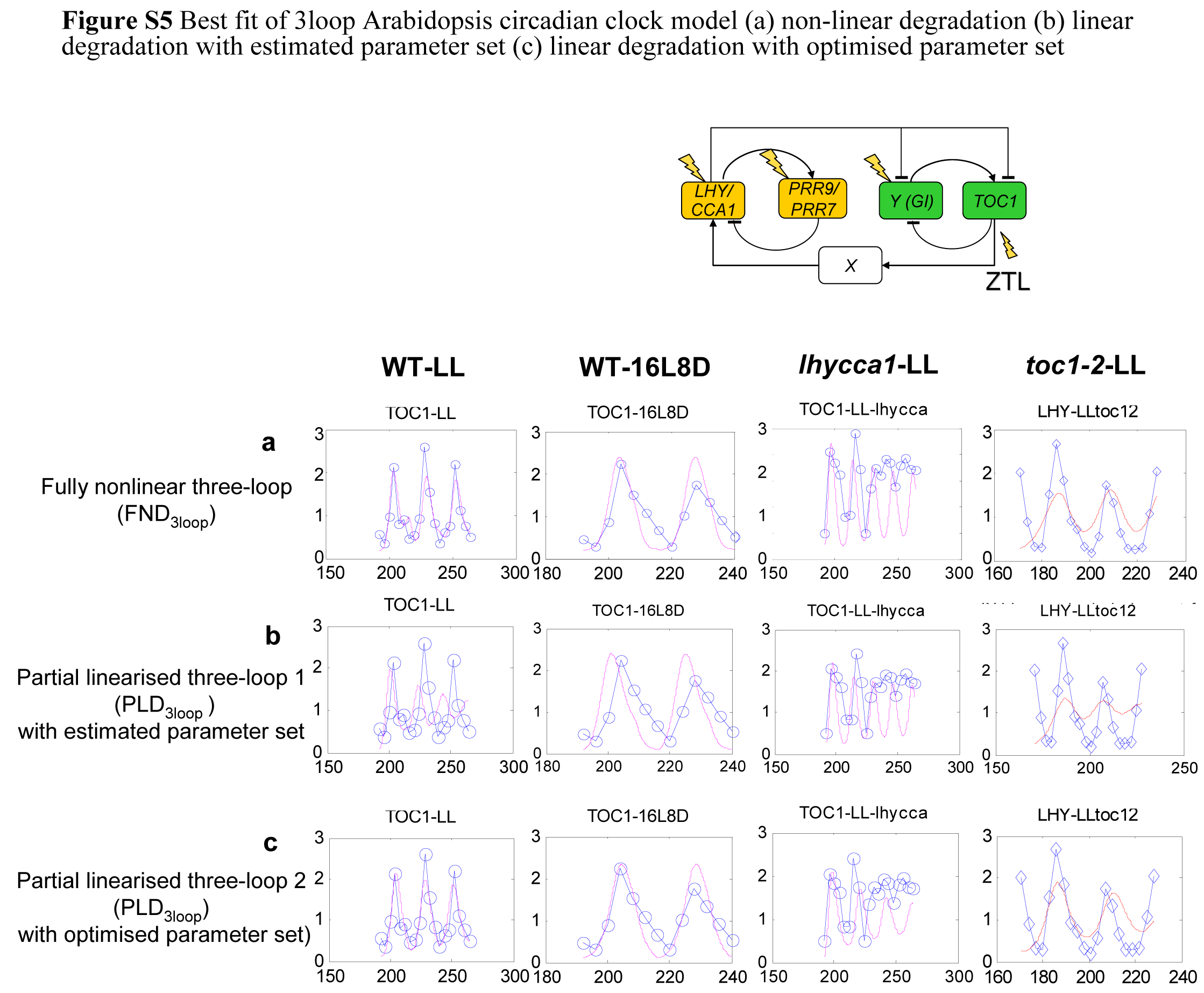

Supplement: Figure S5 — Plots showing the best fit of the three-loop Arabidopsis circadian clock model for (a) fully-nonlinear degradation, (b) partially-linearised degradation with estimated parameter set (the initialising parameter set for parameter searching is determined by the ratio of Vmax and Km in the counterpart nonlinear terms of the fully-nonlinear degradation models), and (c) partially-linearised degradation with optimised parameter set. (1.18 MB TIF) [file pone.0013867.s005.tif]

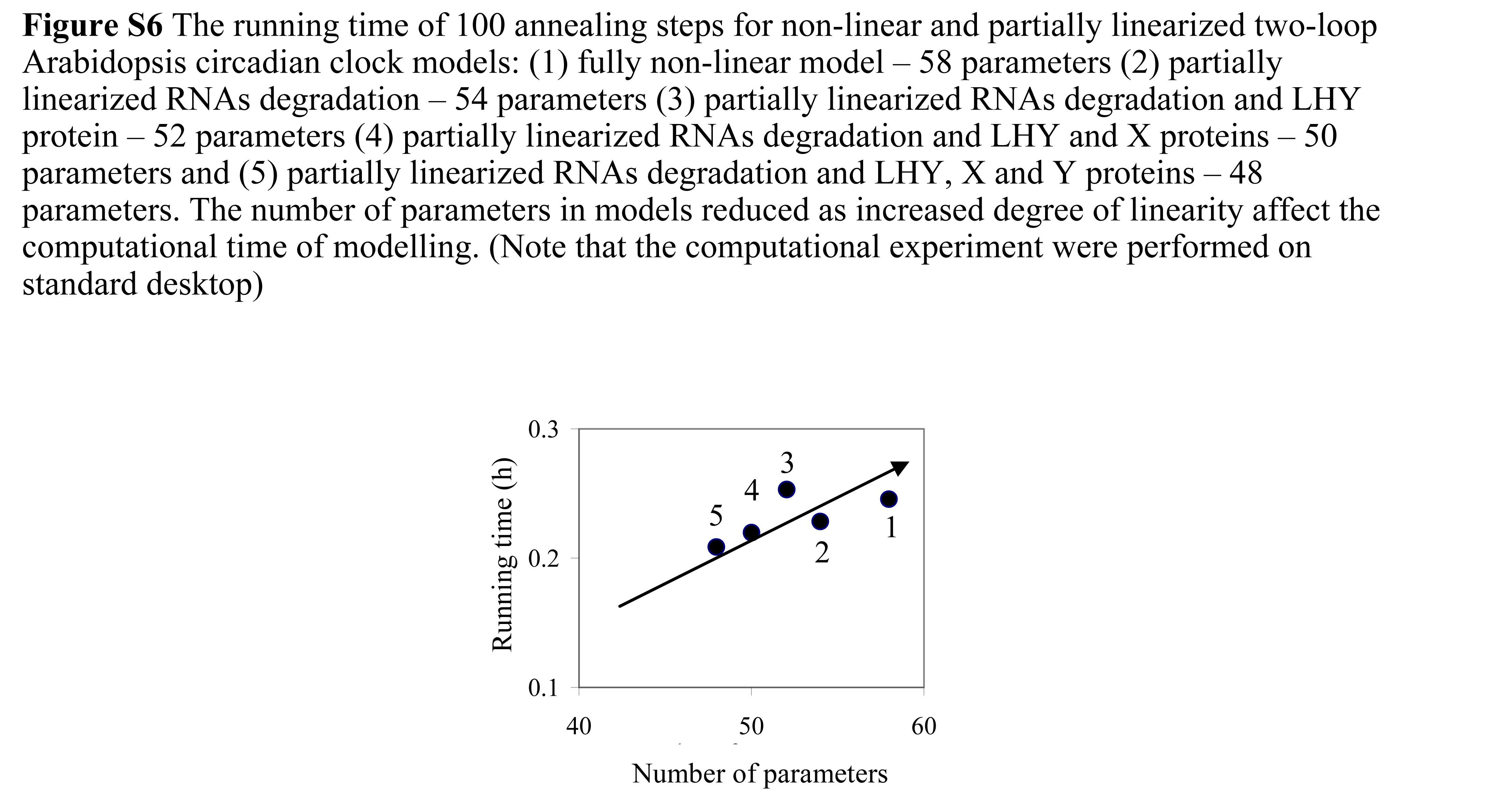

Supplement: Figure S6 — The computational cost of using complex models. The running time for 100 annealing steps (numbers of search cycles through a simulated annealing algorithm) for the nonlinear and partially linearised two-loop Arabidopsis circadian clock models: (1) fully nonlinear model - 58 parameters (2) partially linearised RNA degradation - 54 parameters (3) partially linearised RNA and LHY protein degradation - 52 parameters (4) partially linearised RNA, LHY and X protein degradation - 50 parameters and (5) partially linearised RNA, LHY, X and Y protein degradation - 48 parameters. The number of parameters in models is reduced with increasing degree of linearity, resulting in a reduction of the computational time for the optimisation. Computational experiments were performed on a standard desktop computer with Intel(R) Pentium(R) D CPU 3.00GHz 2.99GHz, 1.99 GB of RAM and Microsoft Windows XP Professional Version 2002 operating system. (1.72 MB TIF) [file pone.0013867.s006.tif]
