# Supplementary material for: The Contributions of Interlocking Loops and Extensive Nonlinearity to the Properties of Circadian Clock Models
Source: PLoS One. 2010 Nov 30;5(11):e13867. doi: 10.1371/journal.pone.0013867 (PMC2994703; doi:10.1371/journal.pone.0013867)
Supplement: Data S1 — The supplementary data consists of model equations and the corresponding sets of parameter used in our analyses. (0.54 MB DOC) [file pone.0013867.s007.doc]

**Model Equations and parameters**

*1. Linearised modified Goodwin models (FLD)*

A linearisation of the degradation rates in the modified Goodwin models (Goodwin, EP and EI) was performed as described in main text. The subsequent systems of ordinary differential equations are given by:

*1.1 Fully linear degradation Goodwin model (FLD_Goodwin_)*

$\frac{dM}{dt}=\frac{V_{M}}{K_{M}+P1^{a}}-V_{\deg,M}\cdot M$ (S1)

$\frac{dE}{dt}=V_{E}\cdot M-V_{\deg,E}\cdot E$ (S2)

$\frac{dP1}{dt}=(V_{P1}+q)\cdot E-V_{\deg,P1}\cdot P1$ (S3)

*1.2 Fully linear degradation EP model (FLD_EP_)*

$\frac{dM}{dt}=\frac{V_{M}}{K_{M}+P1^{a}+P2^{a}}-V_{\deg,M}\cdot M$ (S4)

$\frac{dE}{dt}=V_{E}\cdot M-V_{\deg,E}\cdot E$ (S5)

$\frac{dP1}{dt}=(V_{P1}+q)\cdot E-V_{\deg,P1}\cdot P1$ (S6)

$\frac{dP2}{dt}=(V_{P2}+q)\cdot E-V_{\deg,P2}\cdot P2$ (S7)

*1.3 Fully linear degradation EI model (FLD_EI_):* the model equations are given by Equations S4-S6 for *M*, *E*, and *P1*while the equation of *P2* is given by

$\frac{dP2}{dt}=\frac{(V_{P2}+q)\cdot E}{K_{M}+P1^{a}}-V_{\deg,P2}\cdot P2$ (S8)

$V$ and $V_{\deg}$denote the maximal synthesis and degradation rates of model components, $K$ and $K_{\deg}$ depict kinetic constants of the synthesis and degradation processes, *a*, *b*, and *c* are Hill coefficients and *q(t)* is the light input signal which depends on the time of a day.

Table S1 lists the parameter values of several optimised parameter sets for Fully Nonlinear Degradation (FND) models that gave desired output oscillations, whereas Tables S2, S3 and S4 show parameter values for the corresponding Fully Linear Degradation (FLD) models derived from parameter set1 (Table S1) of Goodwin, EP and EI models, respectively. Note that the *optimised* parameters for linear degradation models are the results of parameter searching through the simulation fit to data. The estimated parameters used to initialise the search were determined by the ratio of *V_max_* and *K_m_* (as described in Equations 9-10 in the main text) of the counterpart reactions in nonlinear degradation models.

**Table S1** Optimised parameter sets for all derived Goodwin models used in this study

| P_ref_ | unit | FND_Goodwin_ | | | FND_EP_ | | | FND_EI_ | | |
| --- | --- | --- | --- | --- | --- | --- | --- | --- | --- | --- |
| Set_ID |  | set1 | set3 | set4 | set1 | set3 | set4 | set1 | set3 | set4 |
| *V_M_* | nM/h | 0.9280 | 0.634 | 0.0939 | 0.9280 | 0.634 | 0.0939 | 0.9280 | 0.634 | 0.0939 |
| *K_M_* | nM | 0.4978 | 0.3109 | 0.65 | 0.4978 | 0.3109 | 0.65 | 0.4978 | 0.3109 | 0.65 |
| *a* |  | 8.0419 | 3.8503 | 7.5385 | 8.0419 | 3.8503 | 7.5385 | 8.0419 | 3.8503 | 7.5385 |
| *V_deg,M_* | 1/h | 0.2350 | 0.4624 | 0.4577 | 0.2350 | 0.4624 | 0.4577 | 0.2350 | 0.4624 | 0.4577 |
| *V_E_* | 1/h | 0.0459 | 0.4527 | 0.5369 | 0.0459 | 0.4527 | 0.5369 | 0.0459 | 0.4527 | 0.5369 |
| *V_deg,E_* | 1/h | 0.2406 | 0.1957 | 0.0665 | 0.2406 | 0.1957 | 0.0665 | 0.2406 | 0.1957 | 0.0665 |
| *V_P1_* | nM/h | 0.3943 | 0.7618 | 0.4939 | 0.3943 | 0.7618 | 0.4939 | 0.3943 | 0.7618 | 0.4939 |
| *V_deg,P1_* | nM/h | 0.2731 | 0.4904 | 0.4175 | 0.2731 | 0.4904 | 0.4175 | 0.2731 | 0.4904 | 0.4175 |
| *K_deg,P1_* | nM | 0.1162 | 0.3168 | 0.2923 | 0.1162 | 0.3168 | 0.2923 | 0.1162 | 0.3168 | 0.2923 |
| *b* |  | 0.4863 | 1.259 | 0.968 | 0.4863 | 1.259 | 0.968 | 0.4863 | 1.259 | 0.968 |
| *V_P2_* | nM/h |  |  |  | 0.2983 | 0.7868 | 0.4327 | 0.0141 | 0.5956 | 0.0344 |
| *V_deg,P2_* | nM/h |  |  |  | 0.4965 | 0.42 | 0.4369 | 0.4704 | 0.5317 | 0.0715 |
| *K_deg,P2_* | nM |  |  |  | 0.8899 | 0.2536 | 0.1214 | 0.8863 | 0.9414 | 0.9964 |
| *c* |  |  |  |  | 2.7700 | 4.2035 | 5.6736 | 4.4254 | 5.0778 | 5.9544 |

**Table S2** Parameter sets for linearised Goodwin models (FLD_Goodwin_) derived from parameter set 1 of the corresponding nonlinear degradation model

| P_ref_ | Description | unit | FND_Goodwin_ (also denoted as Goodwin) | FLD_Goodwin_ (Estimated parameters) | FLD_Goodwin_ (Optimised parameters) |
| --- | --- | --- | --- | --- | --- |
| V_M_ | Max. velocity of component M synthesis | nM/h | 0.9280 | 0.9280 | 0.4844 |
| K_M_ | Michaelis constant of component M degradation | nM | 0.4978 | 0.4978 | 0.0321 |
| a | Hill coefficient of inhibition by P1 |  | 8.0419 | 8.0419 | 9.5577 |
| V_deg,M_ | Max. velocity of component M degradation | 1/h | 0.2350 | 0.2350 | 0.235 |
| V_E_ | Max. velocity of component E synthesis | 1/h | 0.0459 | 0.0459 | 0.2745 |
| V_deg,E_ | Max. velocity of component E degradation | 1/h | 0.2406 | 0.2406 | 0.2406 |
| V_P1_ | Max. velocity of component P1 synthesis | nM/h | 0.3943 | 0.3943 | 0.3363 |
| V_deg,P1_ | Max. velocity of component P1 degradation | nM/h | 0.2731 | 2.3503 | 0.1319 |
| K_deg,P1_ | Michaelis constant of component P1 degradation | nM | 0.1162 | - | - |
| b | Hill coefficient for P1 degradation |  | 0.4863 | - | - |

**Table S3** Parameter sets for linearised parallel-extended Goodwin models (FLD_EP_) derived from parameter set 1 of the corresponding nonlinear degradation model

| P_ref_ | Description | unit | FND_EP_ | FLD_EP_ (Estimated parameters) | FLD_EP_ (Optimised parameters) |
| --- | --- | --- | --- | --- | --- |
| V_M_ | Max. velocity of component M synthesis | nM/h | 0.9280 | 0.9280 | 1.4768 |
| K_M_ | Michaelis constant of component M degradation | nM | 0.4978 | 0.4978 | 0.2011 |
| a | Hill coefficient of inhibition by P1 |  | 8.0419 | 8.0419 | 9.9272 |
| V_deg,M_ | Max. velocity of component M degradation | 1/h | 0.2350 | 0.2350 | 0.2350 |
| V_E_ | Max. velocity of component E synthesis | 1/h | 0.0459 | 0.0459 | 0.9931 |
| V_deg,E_ | Max. velocity of component E degradation | 1/h | 0.2406 | 0.2406 | 0.2406 |
| V_P1_ | Max. velocity of component P1 synthesis | nM/h | 0.3943 | 0.3943 | 0.8585 |
| V_deg,P1_ | Max. velocity of component P1 degradation | nM/h | 0.2731 | 2.3503 | 0.166 |
| K_deg,P1_ | Michaelis constant of component P1 degradation | nM | 0.1162 | - | - |
| b | Hill coefficient for P1 degradation |  | 0.4863 | - | - |
| V_P2_ | Max. velocity of component P2 synthesis | nM/h | 0.0141 | 0.2983 | 1.4014 |
| V_deg,P2_ | Max. velocity of component P2 degradation | nM/h | 0.4704 | 0.5579 | 0.7068 |
| K_deg,P2_ | Michaelis constant of component P2 degradation | nM | 0.8863 | - | - |
| c | Hill coefficient for P2 degradation |  | 4.4254 | - | - |

**Table S4** Parameter sets for linearised interlocking-extended Goodwin models (FLD_EI_) derived from parameter set 1 of the corresponding nonlinear degradation model

| P_ref_ | Description | unit | FND_EI_ | FLD_EI_ (Estimated parameters) | FLD_EI_ (Optimised parameters) |
| --- | --- | --- | --- | --- | --- |
| V_M_ | Max. velocity of component M synthesis | nM/h | 0.9280 | 0.9280 | 0.6398 |
| K_M_ | Michaelis constant of component M degradation | nM | 0.4978 | 0.4978 | 1.3911 |
| a | Hill coefficient of inhibition by P1 |  | 8.0419 | 8.0419 | 2.1086 |
| V_deg,M_ | Max. velocity of component M degradation | 1/h | 0.2350 | 0.2350 | 0.2350 |
| V_E_ | Max. velocity of component E synthesis | 1/h | 0.0459 | 0.0459 | 4.5270 |
| V_deg,E_ | Max. velocity of component E degradation | 1/h | 0.2406 | 0.2406 | 0.2406 |
| V_P1_ | Max. velocity of component P1 synthesis | nM/h | 0.3943 | 0.3943 | 4.6442 |
| V_deg,P1_ | Max. velocity of component P1 degradation | nM/h | 0.2731 | 2.3503 | 2.3159 |
| K_deg,P1_ | Michaelis constant of component P1 degradation | nM | 0.1162 | - | - |
| b | Hill coefficient for P1 degradation |  | 0.4863 | - | - |
| V_P2_ | Max. velocity of component P2 synthesis | nM/h | 0.2983 | 0.0141 | 2.624 |
| V_deg,P2_ | Max. velocity of component P2 degradation | nM/h | 0.4965 | 0.5307 | 0.1322 |
| K_deg,P2_ | Michaelis constant of component P2 degradation | nM | 0.8899 | - | - |
| c | Hill coefficient for P2 degradation |  | 2.7700 | - | - |

*2. Partially linearised two-loop model* *of Arabidopsis circadian clock (PLD_2loop_)*

The published two-loop model of Arabidopsis circadian clock (also denoted as FND_2loop_) [1]was reduced through replacing the nonlinear degradation with linear forms until a large change in desired output was observed. The following system of ordinary differential equations describes a partially linearised two-loop model that gives comparable simulated oscillations to those of the original non-linear model.

(S9)

$\frac{dc_{L}^{(c)}}{dt}=p_{1}c_{L}^{(m)}-r_{1}c_{L}^{(c)}+r_{2}c_{L}^{(n)}-m_{2}c_{L}^{(c)}$ (S10)

$\frac{dc_{L}^{(n)}}{dt}=r_{1}c_{L}^{(c)}-r_{2}c_{L}^{(n)}-m_{3}c_{L}^{(n)}$ (S11)

(S12)

$\frac{dc_{T}^{(c)}}{dt}=p_{2}c_{T}^{(m)}-r_{3}c_{T}^{(c)}+r_{4}c_{T}^{(n)}-\left(\left(1-\Theta_{light}(t)\right)m_{5}+m_{6}\right)\frac{c_{T}^{(c)}}{k_{5}+c_{T}^{(c)}}$ (S13)

$\frac{dc_{T}^{(n)}}{dt}=r_{3}c_{T}^{(c)}-r_{4}c_{T}^{(n)}-\left(\left(1-\Theta_{light}(t)\right)m_{7}+m_{8}\right)\frac{c_{T}^{(n)}}{k_{6}+c_{T}^{(n)}}$ (S14)

(S15)

$\frac{dc_{X}^{(c)}}{dt}=p_{3}c_{X}^{(m)}-r_{5}c_{X}^{(c)}+r_{6}c_{X}^{(n)}-m_{10}c_{X}^{(c)}$ (S16)

$\frac{dc_{X}^{(n)}}{dt}=r_{5}c_{X}^{(c)}-r_{6}c_{X}^{(n)}-m_{11}c_{X}^{(n)}$ (S17)

(S18)

$\frac{dc_{Y}^{(c)}}{dt}=p_{4}c_{Y}^{(m)}-r_{7}c_{Y}^{(c)}+r_{8}c_{Y}^{(n)}-m_{13}c_{Y}^{(c)}$ (S19)

$\frac{dc_{Y}^{(n)}}{dt}=r_{7}c_{Y}^{(c)}-r_{8}c_{Y}^{(n)}-m_{14}c_{Y}^{(n)}$ (S20)

$\frac{dc_{P}^{(n)}}{dt}=0.5\times\left(1-\Theta_{light}(t)\right)-\frac{1.2c_{P}^{(n)}}{1.2+c_{P}^{(n)}}-\Theta_{light}(t)c_{P}^{(n)}$ (S21)

*L,* *T* and *P* denote *LHY*, *TOC1* genes and P-protein while *m*, *c* and *n* indicate molecular entities and their location in the model, i.e. mRNA, protein in cytoplasm, and protein in nucleus, respectively. Θ(*t*) is a step function of light input to the model which is set to be 1 in daytime and 0 in night-time. The additional variables denoted *X* and *Y* represent hypothetical genes *X* and *Y,* respectively. Hill coefficients *b* and *c,* for activation of *TOC1* transcription by *Y* and its repression by LHY, respectively, are constrained to be equal, as they were in the published model. The parameters relating to protein P in (S21) are used to provide transient light input to certain model components. They have not been varied in any of the published models or in the present work. To emphasise this, their labels are replaced in equation S21 by their numerical values (*m15* = 1.2, *k13* = 1.2, *q3* = 1, *p5* = 0.5).

A group of best fit parameter sets is listed in Table S5.

**Table S5** Parameter sets for two-loop models

| P_ref_ | Description | unit | FND_2loop_ | PLD_2loop_  (estimated parameters)* | PLD_2loop_  (optimised parameters) |
| --- | --- | --- | --- | --- | --- |
| q1 | Coupling constant of light activation of LHY transcription | 1/h | 2.5759 | 2.4514 | 2.9028 |
| n1 | Max. light-dependent *LHY* transcription rate | nM/h | 5.1495 | 5.1694 | 3.2767 |
| g1 | Constant of activation by protein X | nM | 0.6717 | 0.6473 | 0.2216 |
| m1 | Max. rate of *LHY* mRNA degradation | nM/h | 1.5912 | 0.8411 | 0.5006 |
| k1 | Michaelis constant of *LHY* mRNA degradation | nM | 1.8528 | - | - |
| p1 | Rate constant of *LHY* mRNA translation | 1/h | 0.8128 | 0.8295 | 0.9573 |
| r1 | Rate constant of LHY transport into nucleus | 1/h | 17.2388 | 16.8363 | 35.6721 |
| r2 | Rate constant of LHY transport out of nucleus | 1/h | 0.1778 | 0.1687 | 0.9402 |
| m2 | Max. rate of cytoplasmic LHY degradation | nM/h | 21.1058 | 13.0657 | 38.8379 |
| k2 | Michaelis constant of cytoplasmic LHY degradation | nM | 1.6292 | - | - |
| m3 | Max. rate of nuclear LHY degradation | nM/h | 3.5018 | 2.8897 | 1.6718 |
| k3 | Michaelis constant of nuclear LHY degradation | nM | 1.2565 | - | - |
| n2 | Max. *TOC1* transcription rate | nM/h | 2.8097 | 3.0078 | 44.0728 |
| g2 | Constant of activation by protein Y | nM | 0.0333 | 0.0338 | 0.0195 |
| g3 | Constant of repression by protein LHY | nM | 0.2894 | 0.2658 | 0.1389 |
| m4 | Max. rate of *TOC1* mRNA degradation | nM/h | 3.9748 | 1.4856 | 2.6789 |
| k4 | Michaelis constant of *TOC1* mRNA degradation | nM | 2.4823 | - | - |
| p2 | Rate constant of *TOC1* mRNA translation | 1/h | 4.2228 | 4.324 | 0.4025 |
| r3 | Rate constant of TOC1 transport into nucleus | 1/h | 0.323 | 0.3166 | 0.1341 |
| r4 | Rate constant of TOC1 transport out of nucleus | 1/h | 1.9641 | 2.1509 | 0.9846 |
| m5 | Max. rate of light dependent cytoplasmic TOC1 degradation | nM/h | 0.0012 | 0.0013 | 0.0002 |
| m6 | Max. rate of light independent cytoplasmic TOC1 degradation | nM/h | 3.2372 | 3.1741 | 0.636 |
| k5 | Michaelis constant of cytoplasmic TOC1 degradation | nM | 2.9809 | 2.7454 | 2.5698 |
| m7 | Max. rate of light dependent nuclear TOC1 degradation | nM/h | 0.0521 | 0.0492 | 0.0256 |
| m8 | Max. rate of light independent nuclear TOC1 degradation | nM/h | 3.897 | 4.0424 | 4.892 |
| k6 | Michaelis constant of nuclear TOC1 degradation | nM | 0.3722 | 0.4033 | 0.8485 |
| n3 | Max. *X* transcription rate | nM/h | 0.2213 | 0.2431 | 0.158 |
| g4 | Constant of activation by protein TOC1 | nM | 0.4181 | 0.4099 | 0.079 |
| m9 | Max. rate of *X* mRNA degradation | nM/h | 9.9471 | 1.5419 | 1.1851 |
| k7 | Michaelis constant of *X* mRNA degradation | nM | 6.5903 | - | - |
| p3 | Rate constant of *X* mRNA translation | 1/h | 2.1317 | 2.147 | 2.0388 |
| r5 | Rate constant of protein X transport into nucleus | 1/h | 1.0439 | 1.0352 | 0.7773 |
| r6 | Rate constant of protein X transport out of nucleus | 1/h | 3.3344 | 3.3017 | 3.8599 |
| m10 | Max. rate of degradation of cytoplasmic protein X | nM/h | 0.2069 | 0.3285 | 0.2159 |
| k8 | Michaelis constant of cytoplasmic protein X degradation | nM | 0.6613 | - | - |
| m11 | Max. rate of degradation of nuclear protein X | nM/h | 3.3455 | 0.1954 | 0.0092 |
| k9 | Michaelis constant of nuclear protein X degradation | nM | 17.9958 | - | - |
| n4 | Light dependent component of *Y* transcription | nM/h | 0.0952 | 0.0854 | 1.5531 |
| n5 | Light independent component of *Y* transcription | nM/h | 0.1673 | 0.1643 | 0.4379 |
| g5 | Constant of repression by TOC1 | nM | 1.1743 | 1.1780 | 0.4907 |
| m12 | Max. rate of *Y* mRNA degradation | nM/h | 4.1362 | 2.4833 | 1.7243 |
| k10 | Michaelis constant of *Y* mRNA degradation | nM | 1.7396 | - | - |
| p4 | Rate constant of *Y* mRNA translation | 1/h | 0.266 | 0.2485 | 0.2152 |
| r7 | Rate constant of protein Y transport into nucleus | 1/h | 2.0966 | 2.2123 | 2.2759 |
| r8 | Rate constant of protein Y transport out of nucleus | 1/h | 0.1866 | 0.2002 | 0.1844 |
| m13 | Max. rate of degradation of cytoplasmic protein Y | nM/h | 0.1195 | 0.0737 | 0.0058 |
| k11 | Michaelis constant of cytoplasmic protein Y degradation | nM | 1.9429 | - | - |
| m14 | Max. rate of degradation of nuclear protein Y | nM/h | 0.5921 | 0.3384 | 0.1963 |
| k12 | Michaelis constant of nuclear protein Y degradation | nM | 1.7554 | - | - |
| g6 | Constant of repression by LHY | nM | 0.0716 | 0.0645 | 0.0540 |
| q2 | Coupling constant of light activation of *Y* transcription | 1/h | 2.4154 | 2.3933 | 1.2198 |
| b | Hill coefficient of activation by protein Y |  | 1.0681 | 1.0258 | 1.9002 |
| c | Hill coefficient ofrepression by protein LHY |  | 1.0681 | 1.0258 | 1.9002 |
| a | Hill coefficient of activation by protein X |  | 3.144 | 3.3064 | 2.56 |
| d | Hill coefficient of activation by protein TOC1 |  | 1.4672 | 1.4422 | 3.1305 |
| f | Hill coefficient of repression by protein LHY for *Y* |  | 1.0588 | 1.0237 | 1.0725 |
| e | Hill coefficient of activation by protein TOC1 for *Y* |  | 3.5161 | 3.6064 | 3.8248 |

* The estimated parameter set is calculated based on the reference parameters obtained from Locke et al., 2005, and used to initialise optimisation that yields the optimised parameter set (see Methods).

*3. Partially linearised three-loop model of Arabidopsis circadian clock (PLD_3loop_)*

The published three-loop model of the Arabidopsis circadian clock (also denoted as FND_3loop_) [2] was partially linearised in the same manner as the two-loop model. The subsequent system of ordinary differential equations is given by:

(S22)

$\frac{dc_{L}^{(c)}}{dt}=p_{1}c_{L}^{(m)}-r_{1}c_{L}^{(c)}+r_{2}c_{L}^{(n)}-m_{2}c_{L}^{(c)}$ (S23)

$\frac{dc_{L}^{(n)}}{dt}=r_{1}c_{L}^{(c)}-r_{2}c_{L}^{(n)}-m_{3}c_{L}^{(n)}$ (S24)

(S25)

$\frac{dc_{T}^{(c)}}{dt}=p_{2}c_{T}^{(m)}-r_{3}c_{T}^{(c)}+r_{4}c_{T}^{(n)}-\left(\left(1-\Theta_{light}(t)\right)m_{5}+m_{6}\right)\frac{c_{T}^{(c)}}{k_{5}+c_{T}^{(c)}}$ (S26)

$\frac{dc_{T}^{(n)}}{dt}=r_{3}c_{T}^{(c)}-r_{4}c_{T}^{(n)}-\left(\left(1-\Theta_{light}(t)\right)m_{7}+m_{8}\right)\frac{c_{T}^{(n)}}{k_{6}+c_{T}^{(n)}}$ (S27)

(S28)

$\frac{dc_{X}^{(c)}}{dt}=p_{3}c_{X}^{(m)}-r_{5}c_{X}^{(c)}+r_{6}c_{X}^{(n)}-\frac{m_{10}c_{X}^{(c)}}{k_{8}+c_{X}^{(c)}}$ (S29)

$\frac{dc_{X}^{(n)}}{dt}=r_{5}c_{X}^{(c)}-r_{6}c_{X}^{(n)}-\frac{m_{11}c_{X}^{(n)}}{k_{9}+c_{X}^{(n)}}$ (S30)

(S31)

$\frac{dc_{Y}^{(c)}}{dt}=p_{4}c_{Y}^{(m)}-r_{7}c_{Y}^{(c)}+r_{8}c_{Y}^{(n)}-\frac{m_{13}c_{Y}^{(c)}}{k_{11}+c_{Y}^{(c)}}$ (S32)

$\frac{dc_{Y}^{(n)}}{dt}=r_{7}c_{Y}^{(c)}-r_{8}c_{Y}^{(n)}-\frac{m_{14}c_{Y}^{(n)}}{k_{12}+c_{Y}^{(n)}}$ (S33)

$\frac{dc_{P}^{(n)}}{dt}=0.5\times\left(1-\Theta_{light}(t)\right)-\frac{1.2c_{P}^{(n)}}{1.2+c_{P}^{(n)}}-\Theta_{light}(t)c_{P}^{(n)}$ (S34)

(S35)

$\frac{dc_{A}^{(c)}}{dt}=p_{6}c_{A}^{(m)}-r_{9}c_{A}^{(c)}+r_{10}c_{Y}^{(n)}-m_{17}c_{A}^{(c)}$ (S36)

$\frac{dc_{A}^{(n)}}{dt}=r_{9}c_{A}^{(c)}-r_{10}c_{Y}^{(n)}-m_{18}c_{A}^{(n)}$ (S37)

*A* denotes APRR7/9 which is an added component of the three-loop model. The parameters relating to protein P in (S34) are used to provide transient light input to certain model components. They have not been varied in any of the published models or in the present work. To emphasise this, their labels are replaced in equation S34 by their numerical values (*m15* = 1.2, *k13* = 1.2, *q3* = 1, *p5* = 0.5).

**Table S6** Parameter sets for three-loop models

| P_ref_ | Description | unit | FND_3loop_ | PLD_3loop_ (estimated parameters) | PLD_3loop_ (optimised parameters) |
| --- | --- | --- | --- | --- | --- |
| q1 | Coupling constant of light activation of LHY transcription | 1/h | 1.7515 | 1.7515 | 3.1251 |
| n1 | Max. light-dependent *LHY* transcription rate | nM/h | 1.3094 | 1.3094 | 1.1272 |
| g1 | Constant of activation by protein X | nM | 0.7409 | 0.7409 | 0.3897 |
| m1 | Max. rate of *LHY* mRNA degradation | nM/h | 1.4779 | 0.4799 | 0.2408 |
| k1 | Michaelis constant of *LHY* mRNA degradation | nM | 3.079 | - | - |
| p1 | Rate constant of *LHY* mRNA translation | 1/h | 0.1868 | 0.1868 | 0.14 |
| r1 | Rate constant of LHY transport into nucleus | 1/h | 6.5186 | 6.5186 | 4.2312 |
| r2 | Rate constant of LHY transport out of nucleus | 1/h | 0.3 | 0.3 | 0.2571 |
| m2 | Max. rate of cytoplasmic LHY degradation | nM/h | 0.0465 | 0.0279 | 0.0214 |
| k2 | Michaelis constant of cytoplasmic LHY degradation | nM | 1.6636 | - | - |
| m3 | Max. rate of nuclear LHY degradation | nM/h | 0.992 | 0.3237 | 0.3325 |
| k3 | Michaelis constant of nuclear LHY degradation | nM | 3.0638 | - | - |
| n2 | Max. *TOC1* transcription rate | nM/h | 31.4464 | 31.4464 | 30.5701 |
| g2 | Constant of activation by protein Y | nM | 8.4771 | 8.4771 | 4.9188 |
| g3 | Constant of repression by protein LHY | nM | 0.2132 | 0.2132 | 0.2929 |
| m4 | Max. rate of *TOC1* mRNA degradation | nM/h | 11.8076 | 11.8076 | 14.3038 |
| k4 | Michaelis constant of *TOC1* mRNA degradation | nM | 6.9263 | 6.9263 | 5.5273 |
| p2 | Rate constant of *TOC1* mRNA translation | 1/h | 4.6918 | 4.6918 | 3.4105 |
| r3 | Rate constant of TOC1 transport into nucleus | 1/h | 12.3939 | 12.3939 | 10.2134 |
| r4 | Rate constant of TOC1 transport out of nucleus | 1/h | 0.0802 | 0.0802 | 0.0643 |
| m5 | Max. rate of light dependent cytoplasmic TOC1 degradation | nM/h | 0.2152 | 0.2152 | 0.154 |
| m6 | Max. rate of light independent cytoplasmic TOC1 degradation | nM/h | 0.1142 | 0.1142 | 0.2089 |
| k5 | Michaelis constant of cytoplasmic TOC1 degradation | nM | 8.4846 | 8.4846 | 7.0835 |
| m7 | Max. rate of light dependent nuclear TOC1 degradation | nM/h | 0.0305 | 0.0305 | 0.0215 |
| m8 | Max. rate of light independent nuclear TOC1 degradation | nM/h | 3.7465 | 3.7465 | 2.8648 |
| k6 | Michaelis constant of nuclear TOC1 degradation | nM | 1.0737 | 1.0737 | 0.6025 |
| n3 | Max. *X* transcription rate | nM/h | 0.2649 | 0.2649 | 0.1396 |
| g4 | Constant of activation by protein TOC1 | nM | 0.0994 | 0.0994 | 0.0535 |
| m9 | Max. rate of *X* mRNA degradation | nM/h | 1.478 | 1.478 | 1.4797 |
| k7 | Michaelis constant of *X* mRNA degradation | nM | 4.5861 | 4.5861 | 3.6202 |
| p3 | Rate constant of *X* mRNA translation | 1/h | 0.6286 | 0.6286 | 1.0779 |
| r5 | Rate constant of protein X transport into nucleus | 1/h | 4.7271 | 4.7271 | 6.0886 |
| r6 | Rate constant of protein X transport out of nucleus | 1/h | 26.6033 | 26.6033 | 13.3817 |
| m10 | Max. rate of degradation of cytoplasmic protein X | nM/h | 0.9189 | 0.9189 | 1.5566 |
| k8 | Michaelis constant of cytoplasmic protein X degradation | nM | 18.0371 | 18.0371 | 12.2952 |
| m11 | Max. rate of degradation of nuclear protein X | nM/h | 2.6171 | 2.6171 | 2.8399 |
| k9 | Michaelis constant of nuclear protein X degradation | nM | 2.5182 | 2.5182 | 3.3879 |
| n4 | Light dependent component of *Y* transcription | nM/h | 0.9055 | 0.9055 | 0.9486 |
| n5 | Light independent component of *Y* transcription | nM/h | 0.6836 | 0.6836 | 0.4579 |
| g5 | Constant of repression by TOC1 | nM | 5.4155 | 5.4155 | 6.1817 |
| m12 | Max. rate of *Y* mRNA degradation | nM/h | 3.4814 | 3.4814 | 4.5857 |
| k10 | Michaelis constant of *Y* mRNA degradation | nM | 0.6615 | 0.6615 | 0.3785 |
| p4 | Rate constant of *Y* mRNA translation | 1/h | 2.5443 | 2.5443 | 4.1583 |
| r7 | Rate constant of protein Y transport into nucleus | 1/h | 2.4917 | 2.4917 | 4.0465 |
| r8 | Rate constant of protein Y transport out of nucleus | 1/h | 1.304 | 1.304 | 0.5135 |
| m13 | Max. rate of degradation of cytoplasmic protein Y | nM/h | 0.8377 | 0.8377 | 0.8729 |
| k11 | Michaelis constant of cytoplasmic protein Y degradation | nM | 0.321 | 0.321 | 0.5416 |
| m14 | Max. rate of degradation of nuclear protein Y | nM/h | 0.0666 | 0.0666 | 0.0327 |
| k12 | Michaelis constant of nuclear protein Y degradation | nM | 14.0005 | 14.0005 | 5.9337 |
| g6 | Constant of repression by LHY | nM | 0.4644 | 0.4644 | 0.721 |
| q2 | Coupling constant of light activation of *Y* transcription | 1/h | 0.2796 | 0.2796 | 0.2291 |
| b | Hill coefficient of activation by protein Y |  | 1.6115 | 1.6115 | 2.8385 |
| a | Hill coefficient of activation by protein X |  | 3.3925 | 3.3925 | 3.426 |
| d | Hill coefficient of activation by protein TOC1 |  | 2.1009 | 2.1009 | 2.2716 |
| f | Hill coefficient of repression by protein LHY for *Y* |  | 1.0682 | 1.0682 | 1.1155 |
| e | Hill coefficient of activation by protein TOC1 for *Y* |  | 1 | 1 | 1.166 |
| n0 | Max. light dependent *LHY* transcription | nM/h | 0.0257 | 0.0257 | 0.0257 |
| n6 | Max. light independent *PRR9/PRR7* transcription | nM/h | 1.21 | 1.21 | 1.21 |
| g7 | Constant of activation by LHY | nM | 0.848 | 0.848 | 0.848 |
| m16 | Max. rate of degradation of *PRR9/PRR7* mRNA | nM/h | 3.6 | 0.4731 | 0.4731 |
| k14 | Michaelis constant of *PRR9/PRR7* mRNA degradation | nM | 7.61 | - | - |
| p6 | Rate constant of *PRR9/PRR7* mRNA translation | 1/h | 21.6 | 21.6 | 21.6 |
| r9 | Rate constant of *PRR9/PRR7* protein movement into nucleus | 1/h | 0.189 | 0.189 | 0.189 |
| r10 | Rate constant of *PRR9/PRR7* protein movement out of nucleus | 1/h | 0.136 | 0.136 | 0.136 |
| m17 | Max. rate of degradation of cytoplasmic protein PRR9/PRR7 | nM/h | 3.69 | 0.1766 | 0.1766 |
| k15 | Michaelis constant of cytoplasmic PRR9/PRR7 protein degradation | nM | 20.9 | - | - |
| m18 | Max. rate of degradation of nuclear protein PRR9/PRR7 | nM/h | 31.8 | 0.9493 | 0.9493 |
| k16 | Michaelis constant of nuclear PRR9/PRR7 protein degradation | nM | 33.5 | - | - |
| α | Constant of repression by PRR9/PRR7 |  | 2.17 | 2.17 | 2.17 |
| q4 | Coupling constant of light activation of *LHY* transcription | 1/h | 3.62 | 3.62 | 2.5782 |
| c | Hill coefficient of repression by Y |  | 2.6851 | 2.6851 | 2.3599 |
| g | Hill coefficient of activation by Y |  | 1.6946 | 1.6946 | 1.6946 |

**References**

1. Locke JCW, Southern MM, Kozma-Bognar L, Hibberd V, Brown PE, et al. (2005) Extension of a genetic network model by iterative experimentation and mathematical analysis. Mol Syst Biol 1: e13.

2. Locke JCW, Kozma-Bognar L, Gould PD, Feher B, Kevei E, et al. (2006) Experimental validation of a predicted feedback loop in the multi-oscillator clock of *Arabidopsis thaliana*. Mol Syst Biol 2: e59.
